# Species aggregation models resolve essential foraging habitat: Implications for conservation and management

**DOI:** 10.1002/eap.70068

**Published:** 2025-07-03

**Authors:** Jarrod A. Santora, Justin J. Suca, Megan Cimino, Elliott L. Hazen, John C. Field, Steven J. Bograd, Brian K. Wells, Isaac D. Schroeder

**Affiliations:** ^1^ Fisheries Ecology Division Southwest Fisheries Science Center, National Marine Fisheries Service, National Oceanic and Atmospheric Administration Santa Cruz California USA; ^2^ Institute of Marine Sciences University of California‐Santa Cruz Santa Cruz California USA; ^3^ Department of Oceanography University of Hawaiʻi at Mānoa Honolulu Hawaii USA; ^4^ Ecosystem Science Division Southwest Fisheries Science Center, National Marine Fisheries Service, National Oceanic and Atmospheric Administration Monterey California USA

**Keywords:** aggregation, conservation, distribution, ecosystem oceanography, foraging habitat, patchiness, realism, spatial organization, species distribution model

## Abstract

Species aggregations are a culmination of behavioral events arising from an array of biophysical interactions, dynamically shifting in space and time. Prediction of species' aggregation dynamics remains a challenge in studies of their distribution patterns. Species distribution models (SDMs) are statistical tools for understanding spatial patterns of marine biodiversity, ranging from essential species habitat, assessing fisheries bycatch, and projecting future distribution changes. SDMs involving pelagic species abundance generally do not typically resolve aggregation patterns. We use a 20‐year observation record of seabird species aggregations, with seabirds being the most easily quantified “pelagic” species, to develop SDMs and a regional ocean modeling system to identify physical drivers and changes in aggregation location and intensity over time. We apply a conceptual ecosystem model to organize environmental covariates according to habitat production within coastal upwelling systems. The SDM used a 2‐step modeling approach: a presence/absence model and a binary aggregation model. Thus, we aim to predict factors that characterize baseline ocean habitat for a species (presence/absence) and that aggregate large numbers of the species. Prediction of seabird aggregation results in realistic spatial distribution patterns that reflect known species habitat associations. Temporally, aggregation indices indicate mixed responses both within and between resident and migrant species, reflecting interannual effects of warm/cool ocean years and mesoscale structure supporting enhanced or decreased productive foraging habitat. The most abundant species were more likely to form aggregations during warmer years, indicating a response to a decrease in productive foraging habitat. The occurrence of species aggregations in spring is predictable by examining ocean‐climate conditions in the preceding winter, thus providing a potential early warning system of anticipated ecosystem shifts. We contend that the aggregation occurrence model may improve the realism of pelagic SDMs and their utility for assessing spatial and temporal variability of trophic interactions. We discuss the utility of species aggregation models for quantifying the variability in critical pelagic habitats, the ecology and response of seabird species as indicators, advancement of ecosystem modeling and monitoring, and conservation applications (e.g., bycatch, wind energy, and oil spills).

## INTRODUCTION

Life in the pelagic ocean is patchy and governed by a triad of biophysical conditions that enhance enrichment, concentrate and retain nutrients, fuel primary production, and promote secondary consumption within trophic hotspots (Bakun, 1996; Santora, Sydeman, et al., 2017). The structure and interaction of life in the pelagic ocean is fluid, spatially organized into a mosaic of ephemeral aggregations, dynamically changing in response to oceanographic variability, biological productivity, and predation intensity. Changes in ocean‐climate conditions can influence production and distribution of prey, resulting in ecosystem shifts that alter the intensity and distribution of predator–prey interactions (Piatt, 1990; Schneider, 1982). Aggregations of mid‐water pelagic prey often beget predator aggregations (Gueron et al., 1996; Mangel, 1994; Okubo, 1986; Parrish & Edelstein‐Keshet, 1999). In coastal upwelling systems, aggregations of forage species are the trophic subunit of ecosystems that structure interactions among predatory fish, seabirds, marine mammals, and ultimately fisheries dynamics (Bertrand et al., 2004; Santora et al., 2020; Scales et al., 2018). Therefore, the prediction of prey and predator aggregations informs where trophic transfer is spatially concentrated and highlights habitat that supports more efficient ecosystem function. Although many forage species occur in aggregations throughout their life cycle for feeding, reproduction, and predator avoidance (Allee, 1927; Brown, 1984), aggregations of their predators are ephemeral by nature and respond to prey availability, ocean conditions that facilitate prey concentration (e.g., fronts; Benoit‐Bird et al., 2019; Mangel, 1994; Okubo, 1986). Furthermore, intra‐ and interspecific associations that may facilitate feeding are important for predator aggregation formation and persistence (Couzin & Krause, 2003; Davoren et al., 2004; Veit & Harrison, 2017). Given the difficulty of observing the complex sequence of biophysical interactions that lead to the formation of predator aggregations, their prediction remains one of the most difficult and elusive objectives in the study of pelagic organisms—and ultimately the spatial organization and function of marine ecosystems (Bakun, 1996).

Marine species aggregations are a culmination of behavioral events arising from an array of biophysical interactions, dynamically shifting in space and time, across a range of spatiotemporal scales. The underlying drivers, and thus the complexity of aggregation dynamics, depend on the scale and scope of the processes examined (Mangel, 1994; Morrell & James, 2008; Parrish & Edelstein‐Keshet, 1999). For example, the location and properties of hydrographic features such as fronts can act as retention mechanisms and thus relate to the structure and concentration of ecosystem productivity (Graham et al., 2001; Rykaczewski & Checkley, 2008; Woodson et al., 2012). These biophysical interactions may range anywhere in scale from a mixed‐surface layer front on copepod aggregations (Wishner et al., 1988), the distribution of mesoscale krill hotspots within upwelling centers (Santora et al., 2011), to shifts in concentrations of anchoveta and fishing vessels within an eastern boundary current system (Bertrand et al., 2004, 2008; Moron et al., 2019) to commercial whaling concentrated along the Southern Boundary of the Antarctic Circumpolar Current (Tynan, 1998). In this study, we focus on predicting the probability of seabird species' aggregations forming within an upwelling ecosystem at spatial scales of fine to mesoscale (10–100 s of km) and temporal scales ranging from days to months (Hazen et al., 2013).

Pelagic species distribution models (SDMs) are statistical tools for understanding the distribution of marine biodiversity, resolving essential species habitat, assessing potential of fisheries bycatch through dynamic ocean management, and projecting future changes in species distribution and the ecosystem services they support (Araújo et al., 2019; Elith & Graham, 2009; Elith & Leathwick, 2009; Gilmour et al., 2022). A review of marine SDMs determined that accounting for aggregation intensity is critically important for understanding and predicting mobile pelagic species distribution patterns (Robinson et al., 2017). Yet, explicitly accounting for aggregation likelihood within predictive SDMs for pelagic species remains difficult (Elith & Graham, 2009; Lee‐Yaw et al., 2022; Sofaer et al., 2019). This in part is due to the data used for developing models which often do not contain information on species abundance or aggregations. For example, many SDMs using bio‐logging data of highly mobile pelagic predators (e.g., seabird, mammal, and large fish) are based on tracked individuals and can only infer the probability of occurrence based on presence‐only information and often the generation of pseudo‐absences (Hazen et al., 2021). When species abundance (or density) and distribution data are available, SDMs may aim to predict both presence/absence and abundance distribution patterns in tandem or separately (Cimino et al., 2020; Muhling et al., 2020; Sofaer et al., 2019; Suca et al., 2022). However, performance of these models indicates better fit for predicting probability of occurrence than abundance, in part due to the difficulty of modeling more complex ecological process such as aggregations (Lee‐Yaw et al., 2022; Shelton et al., 2014; Thorson et al., 2011). Further, predictions of probability of occurrence and abundance patterns often tend to display overly smooth patterns with a lack of high intensity values at ecologically realistic and relevant scales. That is, correlative SDMs may not be capable of predicting aggregation patterns if aggregations are not explicitly considered during model formation. Some mechanistic models, explicitly designed to predict aggregation intensity or mesoscale hotspot patterns (Messié et al., 2022; Santora, Dorman, & Sydeman, 2017), may offer insight on how to incorporate pelagic aggregation patterns into SDMs. However, mechanistic models may require understanding behavioral complexity, substantial parameterization involving life cycle models, and linking coupled ocean‐ecosystem models. Here, we build on previous studies to examine drivers of predator aggregations above and beyond simple presence–absence models.

Marine species aggregations are a result of complex behavioral interactions, involving ocean physics, prey distribution, and interspecies interactions (e.g., exploitative and interference competition and mutualism). Modeling and predicting species aggregations across ecological processes may be considered a behavioral response pathway, but it is difficult and requires many assumptions (Gueron et al., 1996; Mangel, 1994; Morrell & James, 2008; Okubo, 1986). Passive particle‐tracking studies for zooplankton aggregation offer a simple solution for assessing the role of ocean physics in identifying one major process of concentration (i.e., accumulation at fronts), but this approach is often too sensitive to currents and transport dynamics to predict concentrations of upper trophic level organisms (Messié et al., 2022; Santora, Dorman, & Sydeman, 2017). Although some attempts have been made to examine the influence of ocean features, such as Lagrangian coherent structures (e.g., upwelling filaments) within SDMs for predicting presence/absence of individual animals from biologging studies (Scales et al., 2018), predicting species aggregations from physical predictor variables remains a difficult challenge. Therefore, if we focus on observed aggregation patterns to train SDMs, we may better understand which interactive set of oceanographic features most support the presence of species aggregations. These aggregation SDMs could then help identify important ecological areas of trophic interactions (predation/consumption) and further develop strategies for conserving ecosystem function for protected species and fished resources (e.g., marine protected areas).

Here, we focus on developing SDMs using oceanographic model output to simulate seabird species aggregations trained with observations derived from an extensive pelagic ecosystem survey within the California Current Ecosystem (CCE). The CCE is an eastern boundary upwelling system characterized by highly variable and dynamic productivity driven by ocean climate within the North Pacific Ocean with teleconnections to the tropics (Checkley & Barth, 2009; Rykaczewski & Checkley, 2008). Within the CCE, previous particle‐tracking studies involving simulations of an individual‐based model for krill population dynamics, within a coupled regional ocean‐ecosystem model, quantified the formation of krill aggregations and their scales of variability in intensity, size, and area (Santora, Dorman, & Sydeman, 2017). This previous work confirmed the conceptualized biophysical model (i.e., Stommel diagram of plankton aggregations; Haury et al., 1978) describing the scales of variability influencing plankton swarms, or hotspots, pertaining to changes in krill aggregations over days to months, and 10 km to 100 s of km within the CCE. Observations of seabird aggregations within that study provided a geographical assessment of the krill aggregation model's structural realism, furthering our understanding of the scaling, occurrence, and spatial organization of seabird–prey interactions (Santora et al., 2012, 2021). Here, we used 20 years of seabird observations for 6 species to develop an aggregation‐based SDM to predict and simulate aggregation patterns, to learn more about their ephemeral nature and spatial distribution and how their interannual variability may relate to ocean‐climate conditions. These seabird species include a mix of resident breeders (central place foragers) and migrant visitors (trans‐hemisphere and subtropical) species to the CCE. Additional details on seabird species ecology and aggregation are discussed below. Overall, it is our intention that resulting species aggregation models may be used to parameterize future numerical ecosystem modeling studies, aid conservation planning, and inform response and strategy evaluation for oil spills, harmful algal blooms, and associated impacts from fisheries and renewable energy development.

**FIGURE 1:**
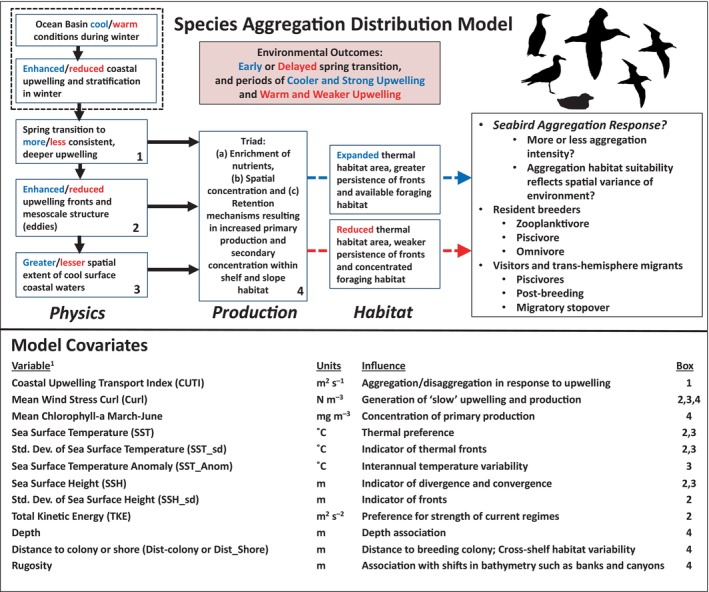
Conceptual overview of the species aggregation model and environmental scope of seabird aggregations as a function of the Triad (enrichment, concentration, and retention processes) as applied to spring/summer ocean conditions within the California Current upwelling ecosystem. The species aggregation model applies mostly physical oceanographic variables derived from a Regional Ocean Modeling System and is organized according to physical drivers that generate productive foraging habitat for seabirds. Although seabird aggregations are likely driven by prey availability and distribution, physical proxies are used in order to make predictions. The spring/summer California Current Ecosystem is influenced by ocean basin‐scale conditions (dashed box) and is characterized by high inter‐annual variability that follows warm and cool ocean conditions that is dependent on conditions during winter and early spring that typically result in early or delayed spring upwelling transition resulting in stanzas of cooler and strong upwelling versus warm and weaker upwelling. Expanded (reduced) cool thermal habitat area and greater (weaker) persistence of fronts occurs during cooler/stronger (warm/weaker) upwelling periods. We examined seabird species aggregation response (e.g., intensity, spatial variance) for a selection of resident and migrant species, and we hypothesized the influence for each variable used in the formation of aggregation models. Model covariates derived and modified from Suca et al. (2022). See Table 1 for generalized species life history information. Taxa silhouettes for common murre, Cassin's auklet were drawn by the author (Jarrod Santora). Silhouettes for the albatross (Alexandre Vong) and the gull and shearwaters (Juan Carlos Jerí) are derived from www.phylopic.org under a CC0 1.0 Universal Public Domain Dedication license (https://creativecommons.org/publicdomain/zero/1.0/).

The three study objectives are to: (1) use a 20‐year observation record of seabird species aggregations to develop aggregation‐based SDMs, (2) predict (hindcast) probability of aggregation occurrence using a regional ocean modeling system to identify physical drivers and changes in aggregation location and intensity over time, and (3) conduct extensive SDM evaluation with independent and lagged oceanographic data to describe scales of variability of seabird aggregations. This latter evaluation step is important because SDMs often overlook the importance of ocean conditions during simulations and ascribe significant difference or capability when it may be lacking (Lee‐Yaw et al., 2022; Sofaer et al., 2019). Importantly, to assist the SDM development, a conceptual ecosystem oceanography model (based on previous studies) is applied to organize model covariates according to the assessment of realistic environmental outcomes that impact habitat production within the upwelling systems (Figure 1). Specifically, our conceptual model examines the “Triad of biological production” (Bakun, 1996), involving environmental covariates that encompass upwelling (enrichment of nutrients), development of upwelling fronts and associated mesoscale features that concentrate biota, and spatial extent of thermal habitat that collectively act to retain primary and secondary production within foraging habitat utilized by seabirds (Santora, Sydeman, et al., 2017). Further, the conceptual model specifically accounts for realistic pathways representing observed environmental outcomes pertaining to years of cool/warm and strong/weak upwelling periods that result in expanded/reduced thermal habitat area, greater/weaker persistence of fronts that affect the extent and quality of foraging habitat. Therefore, we test the overarching hypothesis that the probability of seabird species aggregations reflects ocean‐climate conditions that are dependent on production of suitable foraging habitat. Furthermore, we predict that locally breeding species will aggregate closer to their colonies due to central‐place‐foraging constraints, while aggregations of transient or migratory species will display greater variability in aggregation occurrence.

## METHODS

### Primer on species ecology and aggregations

**FIGURE 2:**
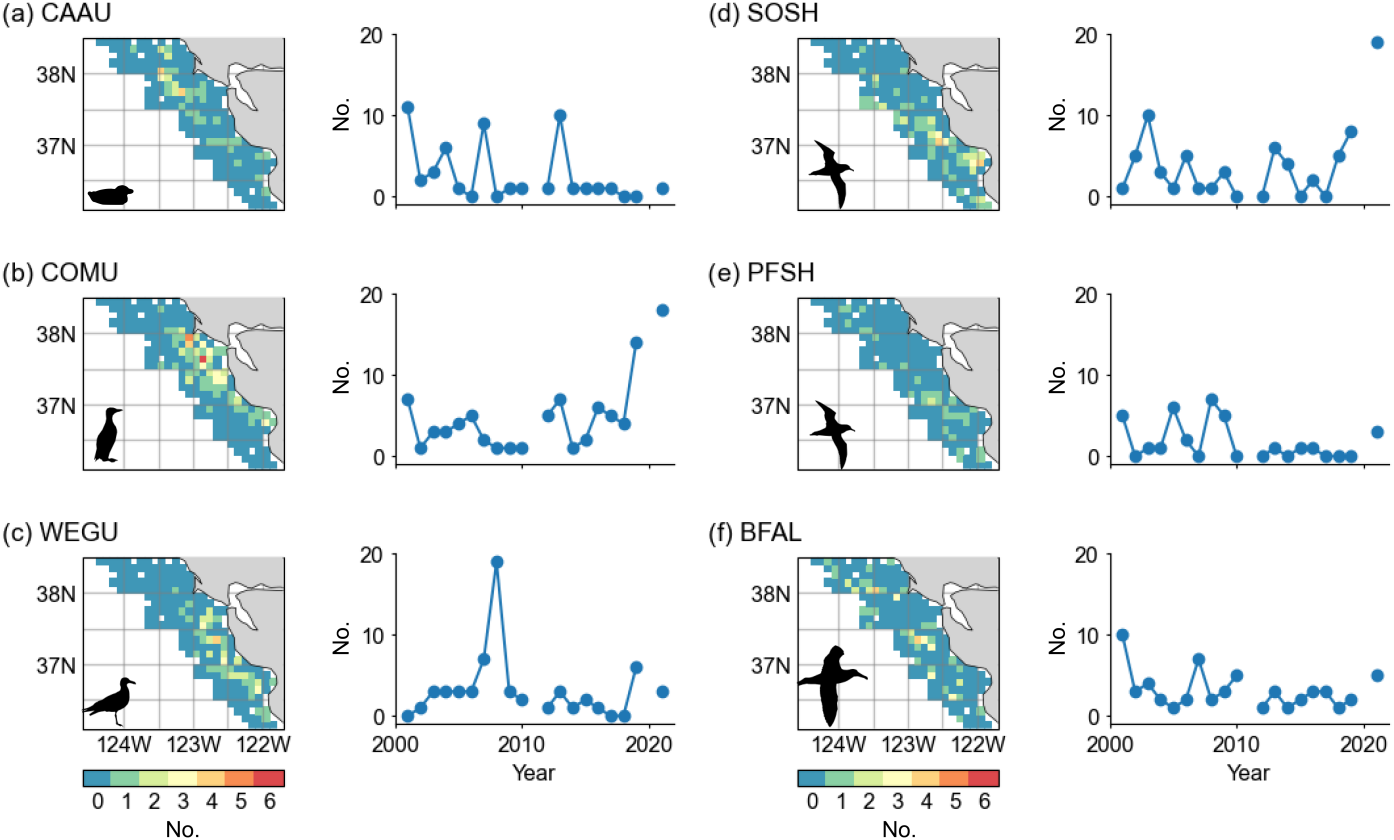
Spatial and temporal observations of total aggregations observed off central California used to train the species aggregation distribution model. Spatial maps represent total number of aggregations per grid cell, reflecting generalized habitat affinities per species. (a–c) Resident breeding species: CAAU, Cassin's auklet; COMU, common murre; WEGU, western Gull; (d–f) seasonal migrant species: SOSH, sooty shearwater; PFSH, pink‐footed shearwater; BFAL, black‐footed albatross. Gaps in in time series are missing years (2011, 2020). Taxa silhouettes for common murre and Cassin's auklet were drawn by the author (Jarrod Santora). Silhouettes for the albatross (Alexandre Vong) and the gull and shearwaters (Juan Carlos Jerí) are derived from www.phylopic.org under a CC0 1.0 Universal Public Domain Dedication license (https://creativecommons.org/publicdomain/zero/1.0/).

We selected several seabird species representing different life history, breeding, and feeding strategies (Figures 1 and 2; Table 1). Cassin's auklet (*Ptychoramphus aleuticus*), common murre (*Uria aalge*), and western gull (*Larus occidentalis*) breed locally within the greater Gulf of the Farallones region, with the largest colonies on Southeast Farallon Island, located in mid‐shelf region of the study area (Ainley & Boekelheide, 1990; Figure 2). Cassin's auklet and common murre (family Alcidae) are pursuit divers that primarily consume a mix of zooplankton and forage fishes (Ainley & Boekelheide, 1990). Auklets generally consume krill and are capable of double brooding in years when the spring transition is early (Ainley & Boekelheide, 1990). Common murre initially forage on krill but switch to juvenile rockfish (*Sebastes* spp.; primarily *Sebastes jordanii*) when they become of sufficient size. Short‐belly rockfish (*S. jordanii*) are usually born in February–March and are spatially ubiquitous in the pelagic waters of the Gulf of the Farallones (Santora et al., 2012, 2014; Schroeder et al., 2009). During lower rockfish recruitment years, murres typically prey‐switch to feeding on northern anchovy (*Engraulis mordax*; Wells et al., 2017; Santora et al., 2021). Murres and auklets breed from March to July and are typically central‐place foragers, making daily trips to foraging locations to procure forage to feed their young (Ainley & Boekelheide, 1990; Warzybok et al., 2018). Some western gulls are year‐round residents, but the majority spend the non‐breeding period along the mainland cost, commencing breeding activity during April–June (Spear, 1988). They are opportunistic foragers that consume a variety of prey (Ainley & Boekelheide, 1990). As primarily visual foragers, gulls also are likely to aggregate in response to the location of other species capable of pursuit diving in search of their prey (e.g., alcids and whales) and may search out locations where other species aggregate in response to prey (Ainley & Boekelheide, 1990; Cimino et al., 2022). In summary, these resident breeding species are most likely to occur in aggregations within proximity to breeding colonies and forage primarily over the continental shelf ranging offshore to slope waters.

**TABLE 1:** Generalized species life history notes for observed seabird species.

Seabird species	Body size, mass	Feeding preference	Diet	Breeding phenology	Breeding location	Conservation status
Cassin's auklet	25 cm, 200 g	Pursuit diving	Plankton and juvenile fish	March–July	Resident of CCE	Near threatened
Common murre	38–46 cm, 900–1000 g	Pursuit diving	Plankton and fish	March–July	Resident of CCE	Least concern
Western gull	55–68 cm, 800–1400 g	Surface feeder and scavenger	Plankton, fish, and squid	March–July	Resident of CCE	Least concern
Sooty shearwater	40–51 cm, 650–978 g	Surface feeder and shallow pursuit diving	Plankton, fish, and squid	November–March	Southern Hemisphere migrant	Near threatened
Pink‐footed shearwater	40–51 cm, 650–978 g	Surface feeder and shallow pursuit diving	Plankton, fish, and squid	November–March	Southern Hemisphere migrant	Vulnerable
Black‐footed albatross	64–74 cm, 2200–4200 g	Surface feeding	Plankton, fish, and squid	February–July	Northern Hemisphere	Vulnerable

*Note*: From: https://www.iucnredlist.org/.

Sooty shearwater (*Ardenna grisea*) and pink‐footed shearwater (*A. creatopus*), Family Procellariidae, are trans‐hemisphere migrants that utilize the California Current as an important feeding habitat during pre‐ and especially post‐breeding phases (Adams et al., 2012; Felis et al., 2019; Spear & Ainley, 1999). When they arrive, adults undergo extensive flight feather molting, which is energetically costly. The breeding phase of both species typically ranges from October–May, but their colonies are located in different regions of the Southern Hemisphere. Pink‐footed shearwater breeds within the Humboldt Current off South America, and sooty shearwaters breed in New Zealand and Patagonia. Both shearwaters have important foraging areas within the CCE and can occur in dense aggregations throughout the region (Adams et al., 2012; Felis et al., 2019). Black‐footed albatross (*Phoebastria nigripes*) are also seasonal migrants to the CCE and breed during November–February on islands in the subtropical North Pacific Ocean (i.e., the Leeward Hawaiian Islands; Arata et al., 2009). Shearwaters and albatrosses are tube‐nosed seabirds that are capable of using olfactory information to find food, especially oil emanating from fish schools (Nevitt, 2008; Reynolds et al., 2005), but all seabirds use visual foraging cues of conspecifics and other species to form aggregations (Hoffman et al., 1981). By comparison with alcids, these species perform dynamic gliding and soaring and are capable of covering broad spatial ranges. Although shearwaters are capable of performing shallow to moderate dives of a few meters to capture prey, albatrosses are restricted to feeding at the ocean surface. These species are also susceptible to fisheries bycatch (Ainley et al., 1981; Forney et al., 2001; Fox et al., 2021; Gladics et al., 2017; Jannot et al., 2021; Takekawa et al., 1990), and several are listed as near‐threatened, threatened, and vulnerable (Table 1).

No other seabird species in the CCE is as abundant as the sooty shearwater (Chu, 1984; Briggs & Chu, 1986; Santora et al., 2012; Spear & Ainley, 1999). The sheer density and occurrence of shearwater aggregations off central California served as the stimulus for the film plot of *The Birds* and are suspected to have been triggered by exposure to toxic algae with wrecks of shearwaters falling on shore, likely exacerbated by their natural tendency to form aggregations (Bargu et al., 2012). Annual estimates of shearwater abundance off California can vary substantially, though prevalence in the CCE appears to have declined (Veit et al., 1997). Common murres are the most abundant resident species and have made a remarkable population comeback (e.g., impacts from egging, oil spill, fisheries bycatch; Ainley & Lewis, 1974; Forney et al., 2001; Warzybok et al., 2018). At their largest colony within the study domain, common murre populations increased from ~50 K in the mid‐1980s to over 200 K in the 2010s (Warzybok et al., 2018). Previous research has demonstrated that at both a broad and meso‐scale, shearwater and common murre aggregations may change as a function of ocean temperature and upwelling conditions (Adams et al., 2012; Ainley et al., 2009; Hyrenbach & Veit, 2003; Wells et al., 2017).

For most marine organisms, their distribution is characterized by patchiness and seabird aggregations are no exception. Decades of visual surveys at sea worldwide have confirmed this reality—many hours or days at sea can pass with few seabird observations and then incredibly dense aggregations, depending on the species, can occur in the hundreds to hundreds of thousands (Davoren et al., 2004; Duffy, 1989; Schneider, 1982; Veit et al., 1997). Species flight behavior and feeding mode, bioenergetics needs, and breeding schedule all collectively influence where a species may aggregate. As creatures of the wind and sea swell, some seabird aggregations can also occur due to a lack of wind, but they are still likely congregating in areas supporting greater availability of prey (Duffy, 1989; Hoffman et al., 1981). For centuries, fishermen have known and relied on the information provided by observing the occurrence of seabird aggregations to identify areas where there were better fishing opportunities. For example, tuna fishery vessels worldwide often use radar in the “bird mode” to look for seabird aggregations many kilometers away to hone in on tuna fishing locations because seabirds are reflective radar targets and associate with tuna schools (Assali et al., 2017). Humans also facilitate seabird aggregations in the form of a response to fishing vessels, especially factory processor vessels, through attraction to fishery discards and baited sets of long‐liners. If not regulated, these aggregative responses to fishing activity generally lead to higher potential bycatch and conservation challenges (Davoren, 2006; Gladics et al., 2017; Melvin et al., 1999).

### Study area and observations

The NOAA Rockfish Recruitment and Ecosystem Assessment Survey (RREAS) is conducted annually during April–June (Julian day 113–163) and monitors pelagic young‐of‐the‐year groundfish abundance and recruitment processes (Field et al., 2021), as well as pelagic biodiversity and ecosystem function within the central and southern CCE. Toward those aims, it uses a combination of survey methodologies and modeling studies (reviewed in Santora et al., 2021). The study area covers the coastal upwelling domain off central California, with transect coverage representing across shelf and alongshore physical and biological conditions. Visual surveys of seabird abundance and distribution are conducted during daylight hours by observers when the vessel is transiting between mid‐water trawling and hydrographic sampling stations (speeds >5 knots). Counts of seabirds are made using standardized strip‐transect methods. Width of the survey transect was estimated with a rangefinder, and seabirds within a strip from the bow and 300 m off the side were recorded (Ainley & Hyrenbach, 2010; Yen et al., 2004). Survey effort is not collected during unfavorable weather, such as low visibility (<300 m) during dense fog, heavy rain, and/or high Beaufort Sea States (e.g., >6). Transects and sightings data were grouped into 3‐km intervals and organized in a publicly available database (https://oceanview.pfeg.noaa.gov/erddap/files/RREAS_FI_SBAS_obs/).

For this study, we use sightings data from 2001 to 2021, bounded by 36–39° N, to match the contemporary satellite and ocean modeling record. Sightings and effort data were gridded on the ocean modeling grid (0.1°× 0.1°; see below), and grid cells with <5% survey coverage were removed; survey years with less than 50 sightings records (3‐km bins) were removed (e.g., 2000); no observations were made in 2011 (due to no available observer) or in 2020 due to the pandemic (Santora et al., 2021; Figure 2). Additional details on observed species distribution patterns and aggregations within this region are summarized in Santora et al. (2012), Santora, Sydeman, et al. (2017), Santora, Dorman, and Sydeman (2017), and Santora et al. (2021). These data are used to monitor temporal changes in seabird species abundance and inform studies pertaining to predator–prey interactions, as well as the evaluation of several numerical ecosystem and SDMs (Cimino et al., 2020; Fiechter et al., 2020; Wells et al., 2017).

The distribution of seabird abundance sightings from at‐sea surveys is typically zero‐inflated and therefore has long tails with higher abundance values usually comprising the top 10% of sightings (Oppel et al., 2012). This reality leads to difficulty for fitting seabird abundance SDMs due to poor performance for predicting localized high abundance areas, resulting in realistic spatial patterns but unrealistic amplitude in abundance (Lee‐Yaw et al., 2022). Application of hurdle model approaches for seabird abundance, through modeling the absences and abundances separately, has led to improvements in predictions but usually does not improve identification of regional hotspots or realistic intensity of species aggregation patterns (Warwick‐Evans et al., 2021). For the purposes of this study, seabird species aggregations are bounded and defined by the resolution of observational data, where counts of individuals are pre‐binned into 3‐km intervals and further aggregated into the ocean modeling grid. During surveys, sightings of seabirds were categorized in three behavioral categories: flying, on the water, and feeding (capturing or pursuing prey at the surface for non‐alcid species). Since we are interested in seabirds engaged in foraging aggregation behavior, all flying birds were removed (despite whether flying to a feeding flock), and counts of sitting and feeding individuals were retained. Across all survey effort, histograms of sightings abundance were made on a per species basis, and aggregation cutoffs were set at the 90th quantile of individuals. This resulted in a simplified distribution of true absences (based on survey effort where the species was not observed) and high abundance aggregations (Table 2; Figure 2). Given the nature and relative rarity of seabird aggregations, this study takes a longitudinal perspective that integrates as much effort as possible over nearly 2 decades (Figure 2).

**TABLE 2:** Summary statistics for observed seabird species aggregations (after applying the 10% threshold) collected during annual Rockfish Recruitment and Ecosystem Assessment Survey; see Figure 2 for spatiotemporal patterns.

Species	*N*	Min (10% cutoff), max, med	Mean ± SD	Total Ind.
Cassin's auklet	62	31, 654, 70	99.97 ± 106.07	9379
Common murre	116	46, 507, 93	119.69 ± 87.22	22,072
Western gull	73	9, 150, 13	23.67 ± 27.11	3126
Sooty shearwater	110	193, 3218, 325	431.97 ± 350.23	73,544
Pink‐footed shearwater	46	8, 129, 12	21.87 ± 21.11	1860
Black‐footed albatross	81	6, 62, 9	10.98 ± 7.92	1941

*Note*: *N* is total count of aggregations; other statistics reflect individuals counted within aggregations.

### Environmental predictors

Daily environmental data were obtained from an assimilative Regional Ocean Modeling System (ROMS) built for the CCE (Neveu et al., 2016). The model contains 42 vertical‐terrain‐following layers from 30 to 48° N and shore to 140° W. Environmental predictors were matched to seabird presence/absence and aggregation locations at the 0.1°×0.1° spatial scale and daily level. We considered a suite of predictors used in previous studies in this region for coastal pelagic species (e.g., krill and squid SDMs; Cimino et al., 2020; Suca et al., 2022): sea surface temperature (SST), sea surface height, wind stress curl (calculated over a 0.5°×0.5° grid), total kinetic energy, SD of sea surface temperature (0.3°×0.3° grid), and SD of sea surface height (0.3°×0.3° grid). The spatial matching scale for SD of sea surface height, sea surface temperature, and wind stress curl was used to generate agreement between a ROMS historical re‐analysis (1980–2010) and a ROMS near real‐time product (2011‐present) (Brodie et al., 2018). Satellite‐derived surface chlorophyll *a* estimates were obtained from the Garver–Siegel–Maritorena (GSM) model from GLOBcolour using level 3 fields at 4 km (Fanton d'Andon et al., 2009; Maritorena & Siegel, 2005). We also used static bathymetric variables derived from the ETOPO1 Global Relief Model: bottom depth and rugosity (SD of depth on a 0.3°×0.3° grid) and computed distance to breeding colony on Southeast Farallon Island and distance to shore.

### Aggregation model framework, evaluation, and prediction

The SDM focuses on predicting the relative likelihood of a seabird species aggregation occurrence. The modeling goals of the study were to: (1) apply machine learning techniques to develop distribution models of seabird species aggregation, using sightings data linked with environmental data to generate daily predictions from 1998 to 2021, (2) evaluate model output and describe spatial and temporal variance to develop aggregation indices, and (3) compare species aggregation indices with spatiotemporal variance estimates derived from independently observed ocean variables from satellite.

We modeled the distribution of seabird species aggregations using a 2‐step modeling approach (hurdle model approach): a presence/absence model and a binary aggregation model (Suca, 2025). The logic behind this approach is that conditions are likely to differ between what allows a given species of seabird to occur at a location compared to what is likely to spur an aggregation. These aggregations, however, occur within the suitable habitat of each species. Thus, predicting an aggregation must factor in both what characterizes baseline ocean habitat for a species (presence/absence) and what aggregates large numbers of the species (aggregation/non‐aggregation). For each observation (uniquely observed grid cell), each species was identified as present/absent and aggregating/non‐aggregating. Aggregations were defined as a density of seabirds in the top 10% quantile of observations where that species was observed (Duffy, 1989; Santora, Sydeman, et al., 2017). A sensitivity analysis of this threshold indicated that results are robust within 5% variability of this cut‐off. According to this definition, the threshold for aggregation varied by species, ranging from 193 birds in the case of sooty shearwaters to only 6 individuals for black‐footed albatross (Table 2). We used this threshold to account for variability in the flock size and aggregating behavior of each species (e.g., observing 6 sooty shearwaters is quite common and does not constitute an anomalous aggregation, while there are no observations of 200 black‐footed albatross in the dataset).

We used boosted regression trees (BRT) (Elith et al., 2008), which combines models or classification trees with stochastic boosting to decrease model variance and increase model performance. Our hurdle approach used a binomial distribution for both stages of the hurdle model (i.e., Pr(Agg) = Pr(1) × Pr(Agg|1)). The first stage defines whether or not a given seabird species was present, while the second defines whether an aggregation occurred (Suca, 2025). This was done to allow for separate inferences regarding the drivers of a species occurrence and their aggregation which are likely to be confounded when simply looking at either problem in isolation. Further, we did not perform a typical hurdle model that accounts for exact abundance in the second stage as we are particularly interested in large, rare aggregations of each species rather than simply their density. For each model stage (presence/absence and aggregation/non‐aggregation), we ran 50 iterations of fourfold cross‐validation (75% of the data was used for training and 25% for testing) (Cimino et al., 2020) and reported the mean output of these 50 models. We used a tree complexity of 3, a learning rate of 0.01 for presence/absence and 0.005 for aggregation models, and a bag fraction of 0.6 (Elith et al., 2008). We report the mean variable importance and response curves as well as the mean area under the receiver operator curve (AUC) and true skill statistic (TSS) for the test dataset for model evaluation, including leave one year out (LOYO) comparisons. Models were fit and predicted using daily data, and the daily output was used to make monthly model averages from April to June. Model predictions, within the ROMS environment bounded by 36–39° N and waters east of 124.7° W, were hindcasted daily and then averaged monthly (April through June) to develop standardized time series of aggregation occurrence within the study area. These daily estimates of aggregation represent the multiplicative result of the mean estimate of each 50 model ensemble (i.e., Pr(1) × Pr(Agg)). Although we model species presence/absence, the results will largely focus on aspects of describing spatiotemporal variability of aggregation probability based on a synthesis of simulations.

Simulations of species aggregation occurrence and independently observed ocean variables were examined using Empirical Orthogonal Functions (EOFs) to identify and describe spatial and temporal variance patterns (discussed below). An assumption of our modeling approach is that environmental predictors are not independent and are both spatially and temporally correlated (as most physical variables are in the pelagic ocean). This assumption is important because the collection of environmental variables that were selected to evaluate potential drivers is likely to act either directly or indirectly on the variability of a species' aggregation occurrence. Thus, this synthesis is a correlation‐based model to illuminate potential ocean drivers within a dynamical ocean modeling environment, to benefit studies of marine species aggregation and spatial organization of marine ecosystems. The SDM and simulation approach involving ROMS are the same as several studies developed for other taxa (e.g., krill, squid, anchovy, turtles, and whales; Brodie et al., 2021; Cimino et al., 2020; Muhling et al., 2020; Suca et al., 2022). Thus, our approach here on identifying scales of variability and comparison to independently observed physics has the potential to improve forecasts of species occurrence and habitat suitability. Further, these previous SDMs were developed using the same variables for prey species (with good performance), so these variables are likely to be useful for predicting predators that consume those prey.

### Independent evaluation and scales of variability

We used EOF analysis, a common signal processing technique, to develop standardized time series of species aggregation occurrences that represent the primary source of variance from the simulations. This technique identifies where the primary variance is likely derived from spatially, thus providing inference on scales of variability. Typically, the first EOF (i.e., component 1) describes the mean spatial and temporal pattern of the signal. We conducted a complementary analysis to determine the robustness of primary variance of the model output described by the EOF analysis. We plotted the spatial average per month of species aggregation occurrence and applied a simplified threshold analysis based on counting the total number of probability values of aggregation that were greater than 2 SDs (hotspot identification; Santora, Sydeman, et al., 2017; Appendix S1: Figure S1a,b). Subsequently, these values were summed per month as an index of the total area of aggregation hotspots (counts of grid cells) and were normalized for comparison with EOF results. Thus, we compared the area of high probable aggregation occurrence with EOF results to evaluate models' performance at describing the temporal and spatial scales apparent for each species aggregation occurrence.

Our second goal for model evaluation involved the comparison of EOFs for species aggregation and independently observed ocean conditions derived from satellites. As previously described, the ROMS applied for aggregation modeling is a data‐assimilative ocean model that incorporates historical and near‐real‐time ocean observations. The objective here is to determine to what degree simulations of species aggregation are correlated with independently observed ocean physics. This is important not only for assessing model realism and communicating uncertainty from a process point of view but also for advancing predictions or incorporating model output within other models. For example, if there are satellite‐based indicators that are correlated with species aggregation model output, then monitoring those variables could provide sources of information on more frequent, consistent, or relevant time scales for ecosystem management. For these purposes and the scope of our case study, we summarized satellite observations of monthly averaged SST and Sea Level Anomaly (SLA), developed EOFs per month, and calculated Spearman rank correlations to assess temporal coherence with species aggregation EOF results. SLA is the difference between actual sea surface height (SSH) and the long‐term mean SSH and reflects the regional extent of anomalous water in the coastal ocean. A positive (negative) SLA is the sea surface that is higher (lower) than average. The SST data are from the Optimal Interpolation SST product (OISST), and SLA data are derived from Aviso (https://www.aviso.altimetry.fr/en/data/products/sea‐surface‐height‐products.html).

Lastly, we assessed linkages between these observed ocean variables and species aggregation occurrence at months preceding the model prediction period (April–June). In the North Pacific Ocean, basin‐scale temperature and ocean transport conditions during winter relate to ocean‐climate indices, such as El Niño/La Niña and Pacific Decadal Oscillation, which are known to influence ecosystem productivity patterns later in spring (Black et al., 2010; Bograd et al., 2009; Checkley & Barth, 2009; Schroeder et al., 2013). Lagged spatial correlation maps were estimated by relating EOF time series of species aggregation to observations from satellite onto grid cells relating to the modeling domain. For example, the EOF time series for a given species in May was spatially correlated with observed SST and SLA values each month sequentially back to January with time series within each grid cell. This evaluation provides inference on potential winter pre‐conditioning effects that may provide leading indicators on when a species might be more or less aggregated during spring. Furthermore, lagged spatial correlation maps can provide an evaluation of whether species aggregation patterns may be broadly or regionally related to observed ocean conditions, thus identifying areas of importance that could serve as monitoring tools for future assessments (Santora et al., 2021; Schroeder et al., 2009).

## RESULTS

### Prediction of aggregation patterns

**FIGURE 3:**
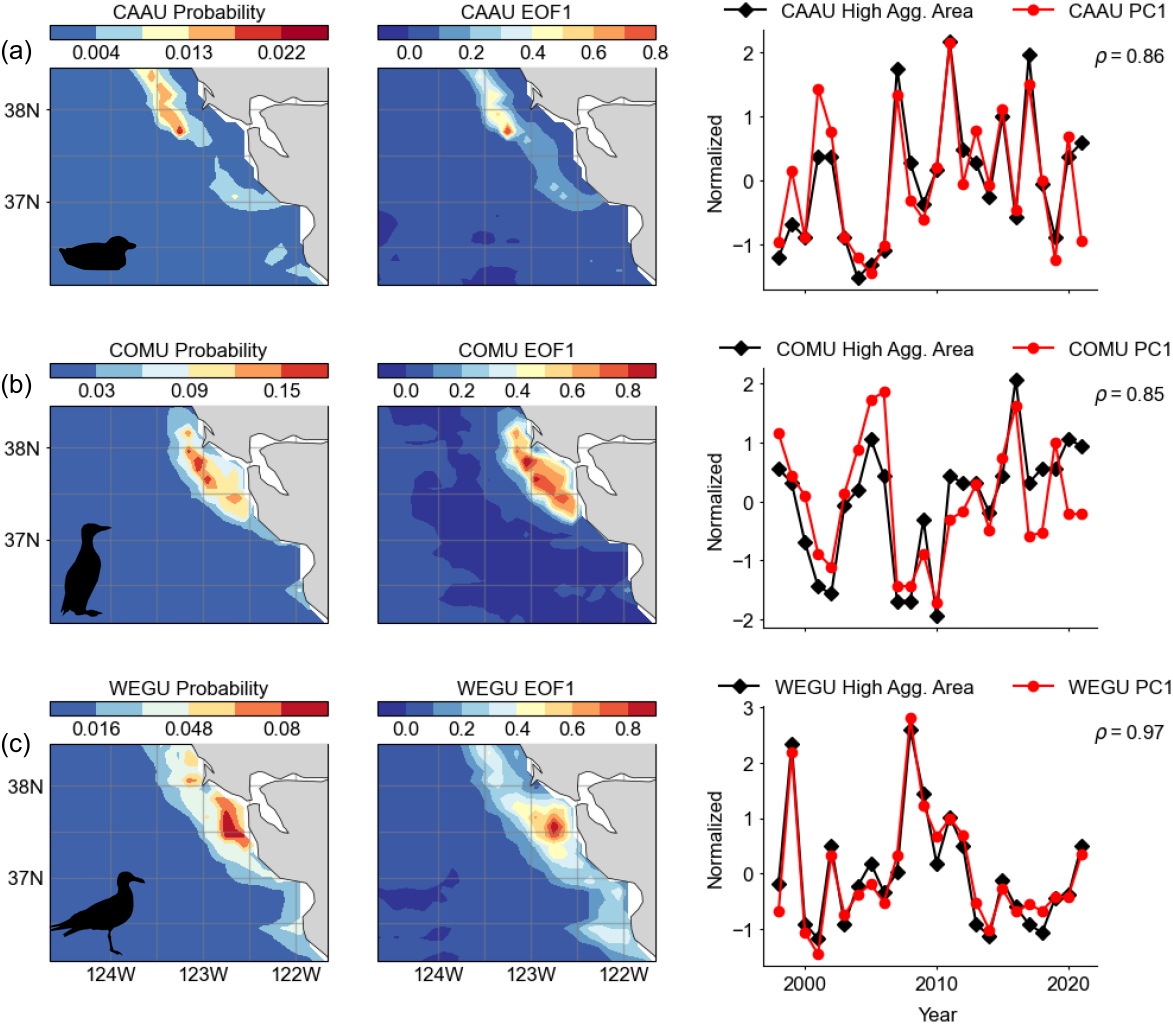
Summarized model output representing spatial mean probability of seabird aggregation, (left) spatial variance pattern of first component of Empirical Orthogonal Function (EOF1), and (right) time series of normalized aggregation index (calculated from threshold analysis of climatology) and the corresponding first principal component (PC1) that represents the temporal signal of the spatial variance of EOF1. (a–c) CAAU, Cassin's auklet; COMU, common murre; WEGU, western Gull; (d–f) SOSH, sooty shearwater; PFSH, pink‐footed shearwater; BFAL, black‐footed albatross. Spearman rank correlation coefficients (ρ) between the normalized aggregation index and PC1 are shown (all significant at *p* < 0.01). Taxa silhouettes for common murre and Cassin's auklet were drawn by the author (Jarrod Santora). Silhouettes for the albatross (Alexandre Vong) and the gull and shearwaters (Juan Carlos Jerí) are derived from www.phylopic.org under a CC0 1.0 Universal Public Domain Dedication license (https://creativecommons.org/publicdomain/zero/1.0/).

**FIGURE 4:**
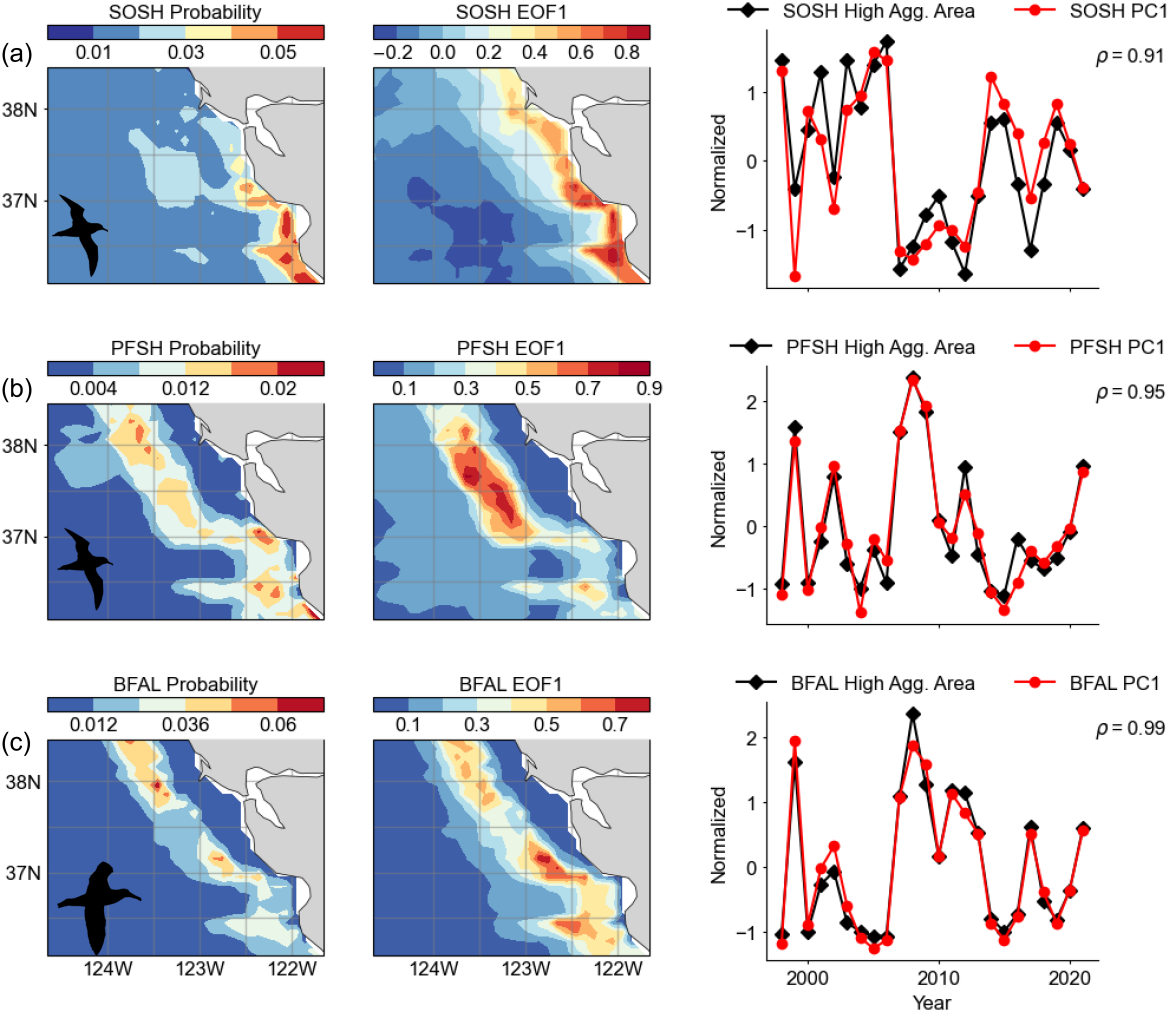
Summarized model output representing spatial mean probability of seabird aggregation, (left) spatial variance pattern of first component of Empirical Orthogonal Function (EOF1), and (right) time series of normalized aggregation index (calculated from threshold analysis of climatology) and the corresponding first principal component (PC1) that represents the temporal signal of the spatial variance of EOF1. (a–c) SOSH, sooty shearwater; PFSH, pink‐footed shearwater; BFAL, black‐footed albatross. Spearman rank correlation coefficients (ρ) between the normalized aggregation index and PC1 are shown (all significant at p < 0.01). Taxa silhouettes for the albatross (Alexandre Vong) and the gull and shearwaters (Juan Carlos Jerí) are derived from www.phylopic.org under a CC0 1.0 Universal Public Domain Dedication license (https://creativecommons.org/publicdomain/zero/1.0/).

Based on cross‐validation, AUC and TSS metrics, the BRTs successfully yielded robust model fits for all species, allowing for predictions of seabird aggregations within the ocean modeling framework (Appendix S1: Table S1; Figures S1–S6). Using a 75/25% split for cross‐validation had better performance metrics than LOYO (75/25 AUC: 0.77–0.89, TSS: 0.53–0.71; LOYO AUC: 0.47–0.78, TSS: 0.25–0.49) with P/A and aggregation models generally performing similarly for each species/cross‐validation type (AUC and TSS values within ~0.1), and the aggregation (hurdle) model performed the best (Appendix S1: Table S1). These statistics indicate that there is important information contained in each year that improves model performance. The approaches for developing normalized time series for quantifying indices of species aggregation resulted in similar indices for describing spatiotemporal variance of aggregation probability. Specifically, the threshold analysis applied to the spatial climatology of mean probability of a species' aggregation occurrence is related to their corresponding monthly EOFs applied to averaged model output (Figures 3 and 4; Appendix S1: Figures S2–S7). The coherence between the threshold‐based and EOF‐based analyses confirms that the approaches are representing similar patterns of spatial and temporal variance for the modeled aggregation dynamics. The advantages of the EOF approach for the aggregation intensity index are that the temporal signal is directly related to the spatial variance signal shown in the map (Figure 3) and thus can be compared similarly to physical oceanographic properties (below). For the latter model evaluation results, the EOF‐based aggregation indices are used to examine temporal coherence with independent satellite observations.

Given the relative rarity of seabird aggregations, especially from observation records that essentially are a snapshot, the probabilities of predicted aggregations are expectedly low. Based on the spatial mean climatology and the application of the EOF, the probability of seabird aggregation occurrence resulted in realistic spatial distribution patterns and reflected known species habitat associations (Figures 3 and 4). The spatial distribution of aggregations of resident breeding species was clustered throughout the continental shelf around the principal colony at the South Farallon Islands (Figure 3). The probability distribution of Cassin's auklet aggregations was highest to the north of the colony along the outer shelf‐break (with one area of particularly high importance), while common murre and western gull aggregations were clustered throughout the continental shelf, reflecting their likely range as residents (i.e., central place foragers). By comparison, the probability distribution of aggregations for migrants was spatially more extensive than that of resident species (Figures 3 and 4). Sooty shearwater aggregations were most likely to occur within Monterey Bay and along the continental shelf, while pink‐footed shearwater and black‐footed albatross aggregation were most likely to occur further offshore along the shelf‐break (Figure 4).

**FIGURE 5:**
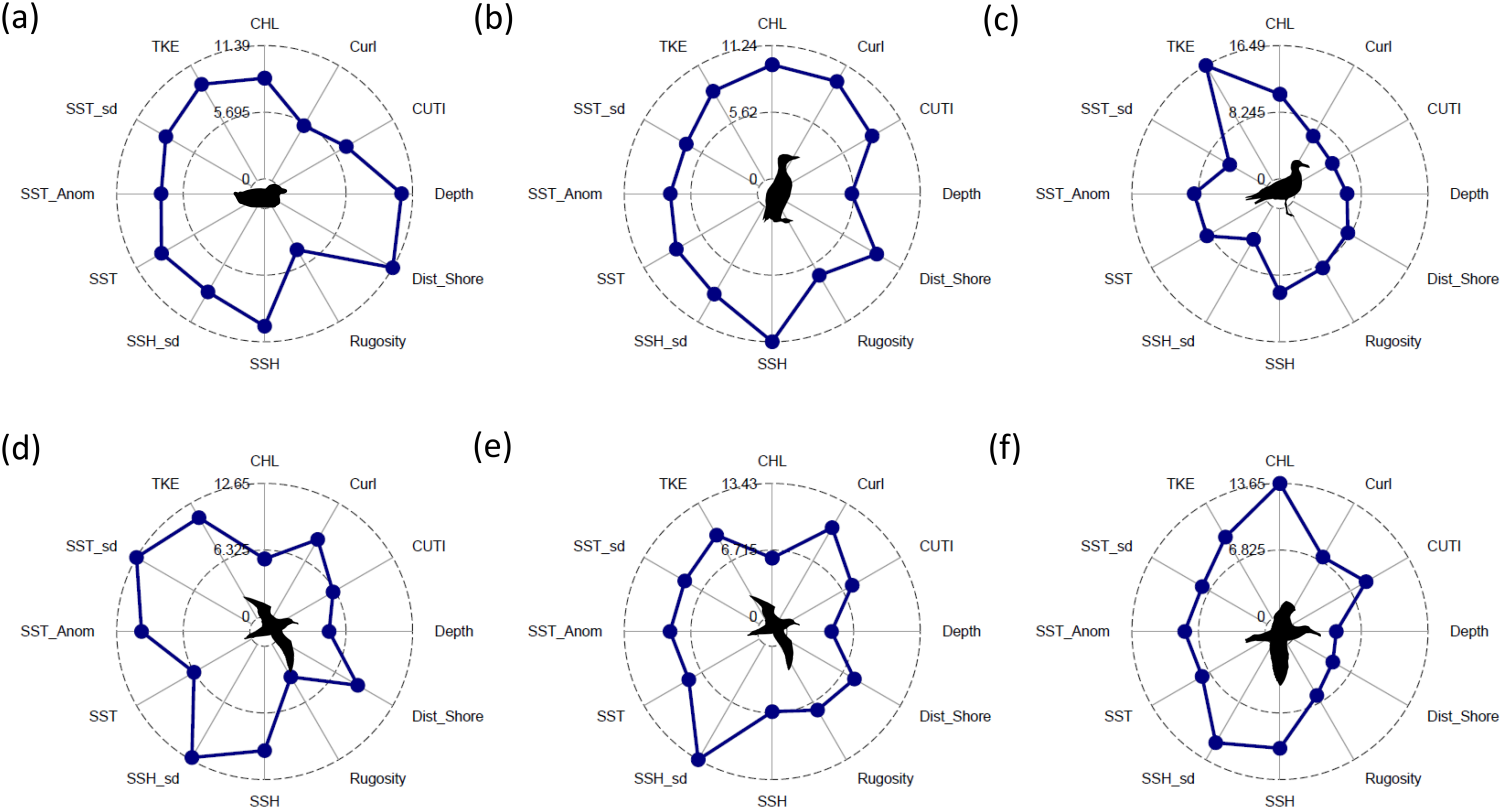
Radar plots representing the relative importance of model covariates for predicting the probability of occurrence of seabird species aggregations: (a) Cassin's auklet, (b) common murre, (c) western gull, (d) sooty shearwater, (e) pink‐footed shearwater, and (f) black‐footed albatross. See Figure 1 for model covariate descriptions. Radar plots for presence/absence models shown in Appendix S1: Figures S2–S7. Taxa silhouettes for common murre and Cassin's auklet were drawn by the author (Jarrod Santora). Silhouettes for the albatross (Alexandre Vong) and the gull and shearwaters (Juan Carlos Jerí) are derived from www.phylopic.org under a CC0 1.0 Universal Public Domain Dedication license (https://creativecommons.org/publicdomain/zero/1.0/).

Although by design, BRT models incorporate the entirety of model covariates for predicting species aggregation patterns, partial dependence plots and supporting radar plots highlight the relative importance of each environmental variable for individual species (Appendix S1: Figures S2–S7). Overall, the species presence/absence models indicated that depth was the most important covariate for 5 of 6 species (except sooty shearwaters), which reflects their general ocean habitat associations (onshore vs. offshore). However, we focus on description of species aggregation model output, noting relative responses with covariates provided by partial dependence plots (i.e., covariates with 9% or greater variable importance). Among resident species, the probability of aggregation for Cassin's auklet was most related to depth, sea surface height, and total kinetic energy; Common murre was related to chlorophyll *a*, wind stress curl, and sea surface height; and Western gull was related to chlorophyll *a*, sea surface height, and total kinetic energy (Figure 5; Appendix S1: Figures S2–S4). For migrants, the probability of aggregation for sooty shearwater was related to curl, sea surface height SD, and total kinetic energy; pink‐footed shearwater was related to sea surface height and SD of sea surface height, sea surface temperature anomaly, sea surface temperature SD, and total kinetic energy; and black‐footed albatross was related to chlorophyll *a*, sea surface height and SD of sea surface height, and total kinetic energy (Figure 5; Appendix S1: Figures S5–S7).

### Inter‐annual variability and independent evaluation

Based on our conceptual model, and a comparison across resident and migrant species, we tested whether seabird species tended to display more or less aggregation response and whether aggregation patterns reflect spatial variance of environmental conditions (Figure 1). Temporally, aggregation indices indicate that there are mixed responses both within and between resident and migrant species (Figures 3 and 4), ultimately reflecting interannual responses regarding effects of warm/cool upwelling variability and mesoscale structure supporting enhanced or decreased productive foraging habitat (Figure 1).

**FIGURE 6:**
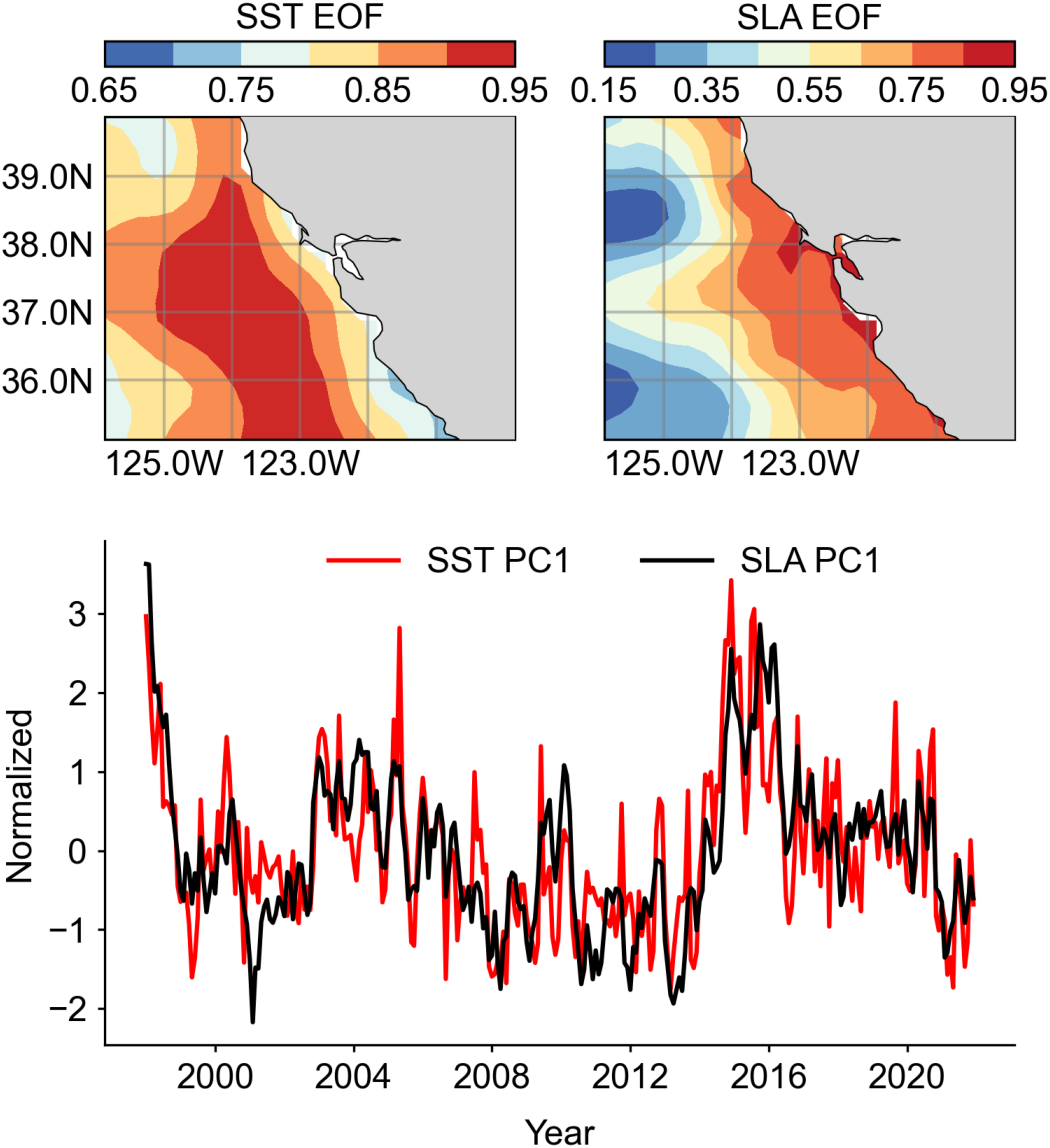
Environmental variables used for independent model evaluation and their spatial and temporal variance described using EOFs: sea‐surface temperature (SST) and sea level anomaly (SLA). These physical variables are not used in the model and derived from satellite observations. SLA is the difference between actual sea surface height (SSH) and the long‐term mean SSH, and it reflects the regional extent of anomalous water in the coastal ocean. (Top) Spatial variance patterns of PC1 and (bottom) normalized time series of PC1 for SST and SLA over January–June.

Of the modeled dynamic ocean variables included in SDMs, SST, and SLA are readily available from satellite products and were considered for developing independent evaluations of SDM output and to assess ocean‐climate impacts on aggregation response. EOF analyses resulted in principal component time series (i.e., PC1) for SST and SLA (from April to June) that are significantly correlated with modeled SST and SSH (Appendix S1: Figure S8). The EOFs for SST and SLA reflect known inter‐annual and decadal temporal patterns of temperature and sea level variability in the CCE (e.g., increases during 2014–2016 heatwave, ENSO years; Figure 5). These spatiotemporal patterns influence the structure and variability of productive foraging habitat used by mid and upper trophic level predators (Figure 1). When ocean conditions are warmer, sea level height is typically higher off California, but spatially, SST is warmer along the outer continental shelf margin (cooler upwelling may persist in the coastal domain), while SLA is higher closer to the coast (Figure 6). Negative SLA and cooler SST coincide with enhanced productive foraging habitat for some species aggregations (Cassin's auklet, western gull, pink‐footed shearwater, and black‐footed albatross).

**FIGURE 7:**
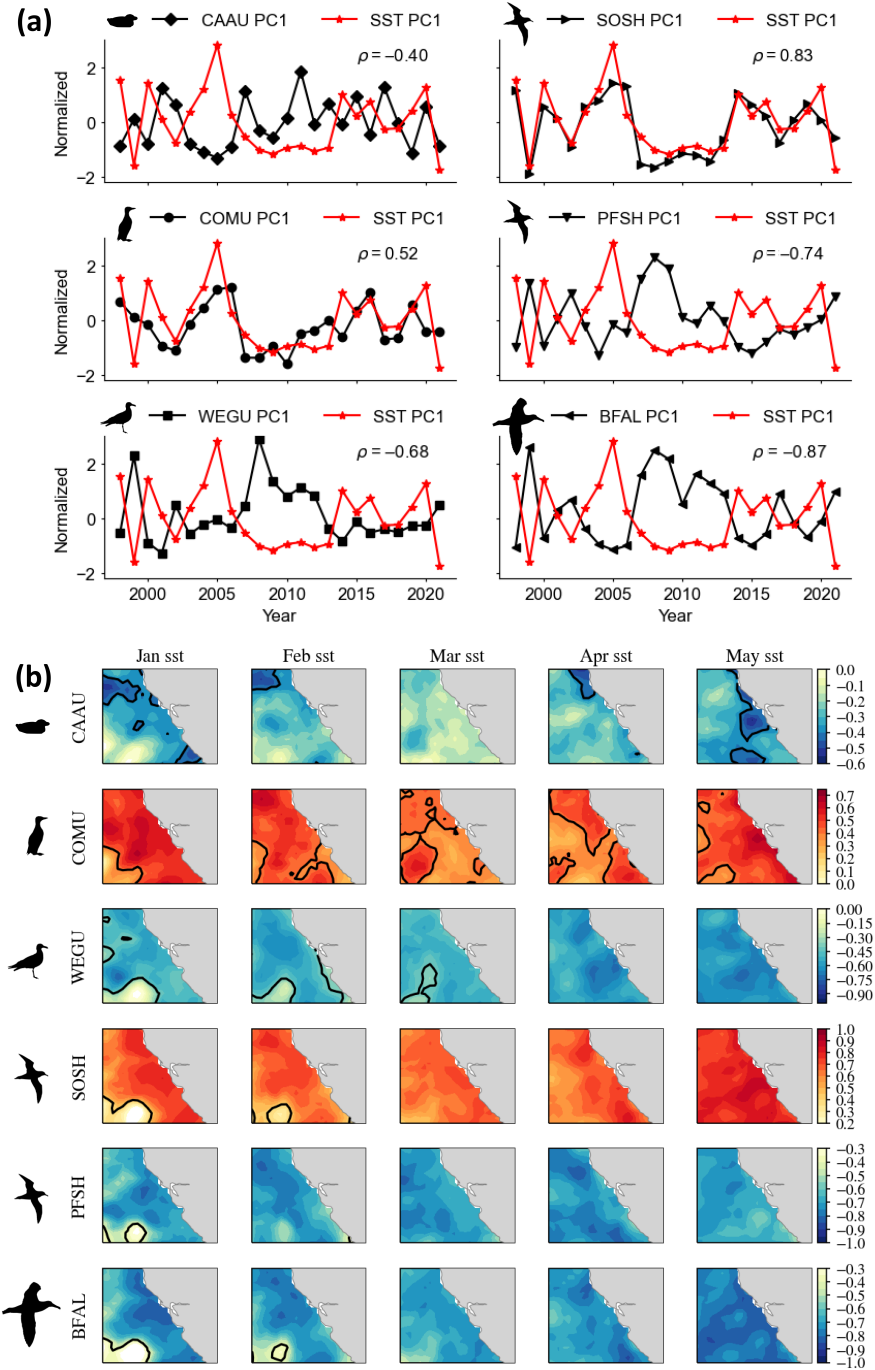
(Top) Spearman rank correlations between the first principal component (PC1) time series for May of the normalized seabird species aggregation index and independent sea surface temperature (SST) not included in the model. All rank correlations are significant at *p* < 0.05. (Bottom) Lagged spatial correlation maps between the May seabird aggregation time series and January–May observed SST. Bold contour lines highlight the area and indicate where correlations are significant at *p* < 0.05. Note that lagged spatial correlation maps indicate broad scale connections with both warm and cool conditions in the north Pacific (except for Cassin's auklet which reflect localized conditions). CAAU, Cassin's auklet; COMU, common murre; WEGU, western Gull; (d–f) SOSH, sooty shearwater; PFSH, pink‐footed shearwater; BFAL, black‐footed Albatross. Taxa silhouettes for common murre and Cassin's auklet were drawn by the author (Jarrod Santora). Silhouettes for the albatross (Alexandre Vong) and the gull and shearwaters (Juan Carlos Jerí) are derived from www.phylopic.org under a CC0 1.0 Universal Public Domain Dedication license (https://creativecommons.org/publicdomain/zero/1.0/).

**FIGURE 8:**
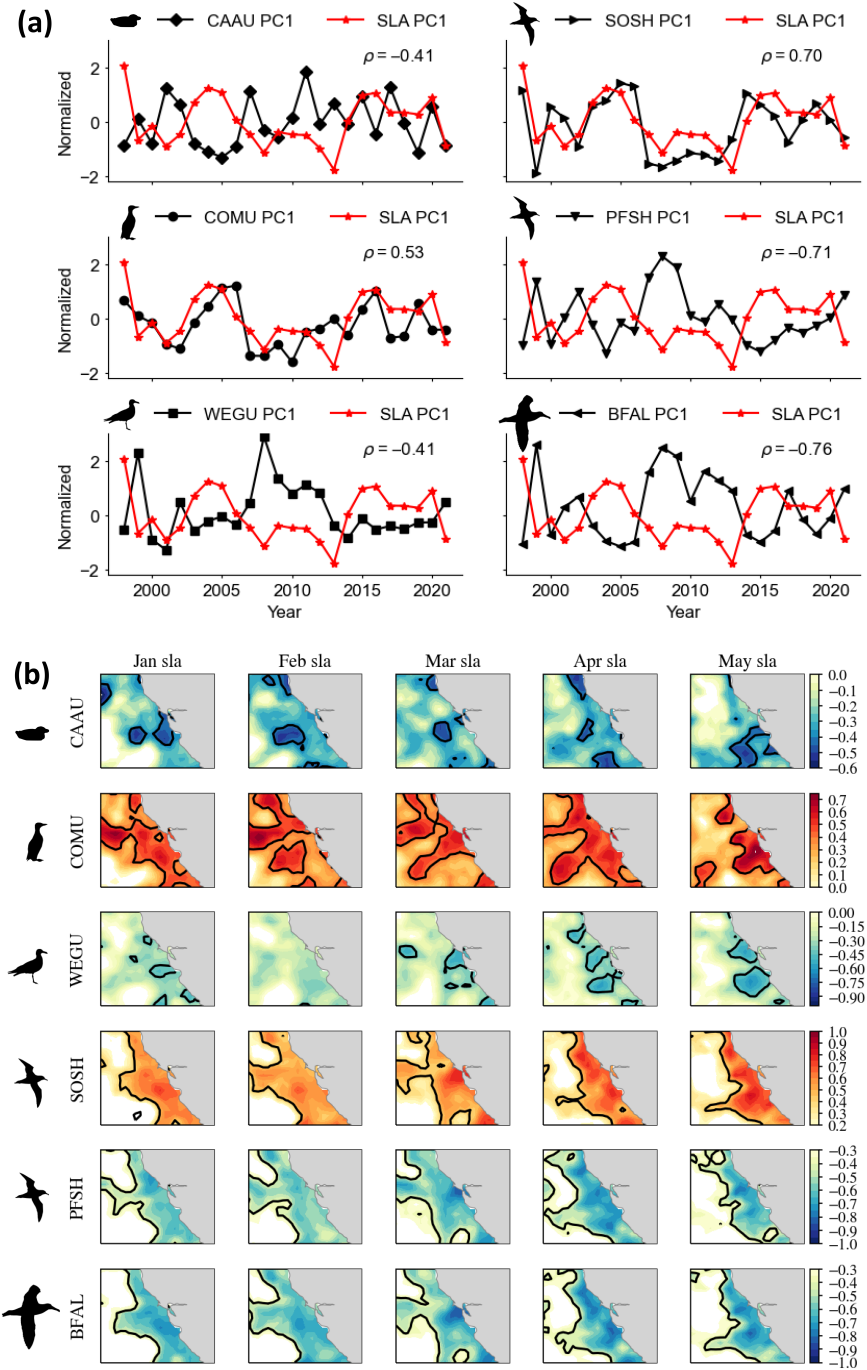
(Top) Spearman rank correlations between first principal component (PC1) time series for May of the normalized seabird species aggregation index and independently observed sea level anomaly (SLA) that was not included in the model. All rank correlations are significant at *p* < 0.05. (Bottom) Lagged spatial correlation maps between the May seabird aggregation time series and January–May observed SLA. Bold contour lines indicate significance (*p* < 0.05) and highlights the area where correlations are significant at *p* < 0.05. Note that lagged spatial correlation maps indicate regional mesoscale connections with sea level anomaly conditions within the California Current. CAAU, Cassin's auklet; COMU, common murre; WEGU, western gull; (d–f) SOSH, sooty shearwater; PFSH, pink‐footed shearwater; BFAL, black‐footed albatross. Taxa silhouettes for common murre and Cassin's auklet were drawn by the author (Jarrod Santora). Silhouettes for the albatross (Alexandre Vong) and the gull and shearwaters (Juan Carlos Jerí) are derived from www.phylopic.org under a CC0 1.0 Universal Public Domain Dedication license (https://creativecommons.org/publicdomain/zero/1.0/).

Seabird aggregation response time series and principal components of SST and SLA (during May) are significantly correlated (Figures 7a and 8a). Aggregations of Cassin's auklet, western gull, pink‐footed shearwater, and black‐footed albatross are most likely to occur when SST is cooler and SLA is lower (negatively associated). Aggregations of common murre and sooty shearwater, the numerically dominant species, are positively associated with SST and SLA, indicating that they display an increased tendency to form aggregations during warmer years (i.e., decreased productive foraging habitat). Since these temporal correlations are derived from EOFs, the spatial variance patterns of ocean conditions therefore influence the spatial organization of seabird aggregations (Figures 6, 7, and 8a).

### Ocean‐climate pre‐conditioning effects on aggregation

As part of our conceptual model, we considered winter pre‐conditioning effects on SDM predicted species aggregation patterns during spring (Figure 1) by conducting lagged spatial correlation maps, comparing aggregation time series with observed SST and SLA measurements. Significant correlations suggest that the effect of winter pre‐conditioning on the seabird aggregation occurrence may be probable 4 months prior to spring observations (Figures 7b and 8b). That is, ocean conditions in winter may serve as a leading indicator of aggregation responses in spring, before resident species commence breeding activities and before migrant visitors arrive in the CCE. All species, except for Cassin's auklet, displayed broad and coherent spatial correlations with SST lagged back to January throughout the CCE domain (Figure 7b). These species spatial lag patterns with SST followed the temporal patterns described for May (Figure 7a). The modeled estimated aggregation response by Cassin's auklet indicated the most local sensitivity to SST conditions in the vicinity of central California where they breed (i.e., localized conditions). Overall, lagged spatial correlation maps between seabird aggregation time series (from May) and SLA exhibited more complex spatial patterns compared to SST (Figures 7b and 8b). Specifically, the spatial distribution of significant correlations occurs within a narrower coastal domain, highlighting the utility of SLA as a mesoscale indicator of seabird aggregation occurrence (Figure 8b). Spatial lag correlations between species aggregations and SLA indicated either enhanced (cooler SST, lower SLA) habitat for increased aggregation occurrence for Cassin's auklet, western gull, pink‐footed shearwater, and black‐footed albatross or reduced foraging habitat for increased aggregation occurrence for common murre and sooty shearwater (Figures 7 and 8).

## DISCUSSION

### Birds of a feather flock together

We show that species aggregation models, conceptualized according to development of suitable foraging habitat for probable occurrence, illuminate the role of ocean modulated productivity patterns for quantifying aggregation dynamics of breeding and migratory seabird species within a coastal upwelling ecosystem. Owing to difficulties in modeling pelagic species density (e.g., under‐ and over‐prediction, lack of structural realism of patchy pelagic life), species aggregation models may offer a solution for identifying critical habitat used by seabirds, at realistic scales relevant for ecosystem functioning since aggregation locations may reflect concentrated trophic exchange between primary and secondary consumers (i.e., forage fish and seabird). Simply, predator species aggregations reflect the unique subset of environmental conditions that support and foster consumption of lower trophic levels. Therefore, the framework and results of the species aggregation models have important implications for the study of marine ecosystem functioning and species interactions and subsequently the utility of aggregation information as context to benefit conservation planning and ecosystem management.

In a seemingly featureless ocean environment (to humans), surface discontinuities in hydrographic properties (fronts and eddies) are important foraging cues for visual predators like seabirds (Schneider, 1982, 1991). Due to the role of ocean physics in concentrating production and predators, the species aggregation model developed here is by design a simplified environmental model with little biological complexity, aside from remotely sensed chlorophyll *a*. Our conceptual framework for modeling aggregation occurrence focused largely on physical variables to characterize differences in the annual production of suitable foraging habitat as a function of an environmental triad of biophysical processes (e.g., enrichment, concentration, and retention; Figure 1). Reduction in cool thermal habitat and shoreward shifts may compress available predator foraging habitat to within a narrow band along the coast, thus imposing a concentration effect on pelagic species distribution (Benson et al., 2002; Santora et al., 2020). Species aggregation occurrence patterns reflected the enhancement and reduction of foraging habitat, yet the nature of the response varied among resident and migrant species (discussed further below). Our results indicate that simplified aggregation models using mostly physical ocean variables provides an improved basis for predicting species aggregation occurrence.

Above all, we highlight that the aggregation occurrence model may improve the realism of pelagic SDMs and their utility for assessing spatial and temporal variability of trophic interactions. More case studies, across different species and life histories, would help resolve the potential of the aggregation occurrence approach. We acknowledge that aggregations in reality are density‐dependent processes related to species interactions within suitable environmental conditions (Gueron et al., 1996; Mangel, 1994; Morrell & James, 2008; Okubo, 1986). However, in our framework, aggregations were specified as probability of occurrence (P/A) derived from applying a threshold classification from observational data from standardized surveys and reported literature (Duffy, 1989; Piatt, 1990; Schneider, 1982; Suryan et al., 2012). This simplification was a necessity given the relative rarity of large aggregations. It is known that seabird flocks are dynamically changing in size from minutes to days to months (Hoffman et al., 1981; Schneider, 1991; Veit & Harrison, 2017). Our aggregation index reflects a likelihood of occurrence at the daily scale, but when summarized over a month over decades, it indicates essential seabird foraging habitat that supports aggregations. Admittedly, existing SDM approaches for some marine species that live their entire lives within aggregations may already sufficiently account for aggregation dynamics although the SDM does not explicitly account for aggregations. For example, P/A distribution models of oceanic dolphin species, which occur in remarkably dense aggregations (1000s of individuals), may capture the structural realism of a patchy ocean environment (Becker et al., 2020), but model performance might be improved if thresholds are applied to counts of group sizes, to potentially identify essential foraging habitat (Szesciorka et al., 2023). The SDM aggregation approach may benefit studies of aggregation patterns of terrestrial ungulates where count data and aggregation size might be logistically easier to measure and monitor (Torney et al., 2018), compared to marine mammals within oceanic environments (Becker et al., 2020).

We contend that habitat supporting seabird species aggregations is essential for marine ecosystem functioning because these locations are where trophic interactions, consumption of prey, are most concentrated (Ainley et al., 2009; Bakun, 1996; Santora, Sydeman, et al., 2017). Such areas are likely more persistent within the seascape and may be considered localized predation pits that support significantly higher trophic transfer and contribute to regional seabird demographic population dynamics (e.g., expansion and establishment of new breeding colonies) and important migratory habitat for seasonal visitors. Therefore, we present below a generalized discussion on how species aggregation models can be improved (realism assessments), may be utilized for the study of climate perturbations and ecosystem shifts, and may be used for conservation planning and ecosystem monitoring issues, ranging from fisheries bycatch, renewable energy and development, and response to harmful algal blooms and oil spills.

### Limitations and behavioral needs for modeling aggregations

The aggregation distribution model is an oversimplification of seabird foraging ecology because, in reality, the interaction of seabird species with different life histories and behavior is critical to the formation and persistence of feeding flocks (Duffy, 1989; Hoffman et al., 1981; Veit & Harrison, 2017). The model does not account for the presence of additional species aggregations as predictor of a species' aggregation probability. Although we did not investigate multispecies aggregations, we understand that cooperation and other interactions may drive the formation and persistence of seabird aggregation dynamics (e.g., Ainley et al., 2009). For example, different foraging behaviors of planktivorous and piscivorous seabirds, involving a combination of pursuit diving and surface feeders, may cooperatively work together to concentrate small patches of forage species that may facilitate greater aggregation intensity of multiple species (Veit & Harrison, 2017). Although accounting for the behavioral rules of multi‐species aggregations increases model complexity (Mangel, 1994; Morrell & James, 2008), future prediction of multi‐species aggregations within SDMs could be resolved using either joint SDMs (Arimitsu et al., 2023; Roberts et al., 2022) or perhaps focusing on screening observational survey data for the frequency of occurrence of multispecies aggregations and attempting to model them as demonstrated in this study.

It is currently unclear whether shifts in seabird aggregation occurrence impact the reproductive success of local seabird populations (i.e., chicks fledged, survival, and later recruitment). Major breeding failures of Farallon Island seabird populations have occurred during strong El Niño events and delayed spring upwelling—coinciding in a substantial reduction of available forage species within the foraging halo of the colony (Ainley et al., 1996; Ainley & Boekelheide, 1990; Santora et al., 2014; Wells et al., 2017). In most years, breeding success of common murre is remarkably stable, owing to their flexible foraging strategies and being strong fliers and deep‐divers (Ainley & Boekelheide, 1990; Warzybok et al., 2018). It is not unreasonable to presume that increased aggregations, both in occurrence and size, are a potential murre foraging strategy for finding and locating patchy prey during warm years as a means to benefit and stabilize the breeding performance of the colony. In fact, aggregation is more prevalent during food‐lean years (Ainley & Boekelheide, 1990). Through local enhancement processes (Ward & Zahavi, 1973), seabird aggregations reflect areas where foraging is successful, and in other systems, communication and foraging strategies displayed by murres are readily apparent and linked to changes in forage availability (Davoren et al., 2004). Although group behavior is critical to aggregation formation (Gueron et al., 1996; Morrell & James, 2008), more information is needed to resolve how individuals detect and respond to aggregation occurrence. Satellite‐tracking of breeding western gulls (from the Southeast Farallon Island) indicates that while breeding performance did not vary significantly (gulls are optimal opportunists), the foraging distribution of gulls shifted shoreward during warmer years, with more foraging effort closer to land, potentially in response to shifts in humpback whale distribution (Cimino et al., 2022). Further comparison of species biologging data from focal colonies with aggregation model products may help improve our understanding of behavioral processes and interactions and resource utilization by organisms that live within or form ephemeral aggregations.

### Implications for realism of SDMs

Apparently, based on the literature review of SDMs, probability of occurrence models has better performance than abundance‐based SDMs (Lee‐Yaw et al., 2022). In pelagic systems, this performance difference is undoubtedly challenged by dynamic biological patchiness within a fluid environment. Our hybrid approach here—modeling aggregation occurrence—applied to seabird ecology, provides an opportunity to discuss the potential realism, accuracy, and generality of species‐aggregation models (Araújo et al., 2019; Sofaer et al., 2019). Our seabird‐aggregation model output appears realistic and accurate in the sense that the probability of occurrence increased within the study region and time period and reflected species responses to anomalous thermal conditions that were observed and described previously (Santora et al., 2014; Santora, Dorman, & Sydeman, 2017; Wells et al., 2017). We cannot fully judge the model's ability to predict outside the study time period, but since the observations used to develop the model are derived from a long‐term ecosystem survey, we have the opportunity to use the model to make predictions before future surveys and apply the model to develop and test different survey strategies (Santora et al., 2021). However, our model skill decreased slightly when predicting out‐of‐sample years, which is common for SDM approaches (Brodie et al., 2021; Elith & Graham, 2009; Muhling et al., 2020). This indicates the need for further evaluations with independent observations. Within the study period, the model predicted patterns in a few missing years when sightings data were not available (2011, 2020), with the patterns in those years reflecting the environmental conditions observed in other similar years. Having observations of species aggregations across nearly 2 decades, combined with ocean modeling, likely facilitated a realistic estimate of the mean occurrence of aggregations through space and time (Araújo et al., 2019; Sofaer et al., 2019).

Importantly, model output was evaluated and compared to independent empirical environmental data (Araújo et al., 2019; Elith & Leathwick, 2009), indicating the presence of significant covariation found between species aggregation indices and environmental conditions. This evaluation step highlights a potential conundrum and raises the question of whether marine‐based SDM approaches, which often indicate that SST is a primary factor on model performance, are perhaps too sensitive to SST variability and are reflecting the strongest interannual signal in the collection of environmental covariates (Brodie et al., 2021). Conversely, SST is an important variable for good reason, as many pelagic species do exhibit thermal preferences or respond indirectly to temperature shifts that impact their prey (Ainley et al., 1996; Bertrand et al., 2004, 2008). Bottom depth is usually an important factor in pelagic SDMs (Robinson et al., 2017), as found here for species aggregation occurrence, for identifying species with a preference for nearshore and offshore habitat. The conundrum regarding SST and marine SDM performance may exist simply because temperature displays large spatiotemporal autocorrelation patterns such that the prediction of species probability of occurrence is inflated due to suitable habitat being much larger than it actually may truly be. For example, SST is spatially auto‐correlated in the alongshore direction for 100 s of km (or possibly 1000 s), while smaller features, such as ephemeral fronts and eddies, are structured at finer spatial scales (10 s of km) (Bakun, 1996). Aggregation occurrence is likely a response to the array of environmental conditions occurring at finer spatial scales that modulate the quality of the foraging habitat, ranging over daily to monthly scales within the study region (Figure 1).

Given the uniqueness of the study region in terms of oceanographic conditions (e.g., upwelling, circulation), bathymetric habitat, and forage species, we do not expect that this particular species aggregation model can be generalized to other regions within the California Current (i.e., ocean physics may be different). However, the approach of modeling seabird aggregations, combined with regional ocean modeling systems or satellite data, may be general enough to predict responses of other species in regions of the North Pacific (e.g., Bering Sea). Sooty shearwaters and common murre are ubiquitous species in the North Pacific Ocean, and aspects of their ecology are often used as valuable ecosystem indicators (e.g., Cairns, 1987). At‐sea sightings data of these species (and many others) are available and organized in multi‐decadal datasets of at‐sea seabird abundance that may be used to develop a generalized model of their aggregation occurrence throughout different marine ecosystems in the North Pacific. Ultimately, the aggregation model framework provides a research tool to investigate hypotheses for benefiting the study and prediction of aggregation patterns of highly mobile species (Hazen et al., 2013). The basic premise of the SDM approach tells us where species should be distributed but can now easily be extended to prediction of how likely a species would be aggregated.

Prediction of seabird aggregations not only highlights important foraging habitat for seabirds but also provides information regarding how trophic interactions may be distributed in marine ecosystems. Since aggregations are the subject, this line of study may find a path toward prediction of spatially explicit consumption patterns to better parameterize resource needs of predators within ecosystem models. This would allow assessment of tradeoffs among changes in environmental conditions that alter aggregation intensity and predation pressure on managed living resources (e.g., coastal pelagic species). For example, species bioenergetics models are available within the study region (developed from diet studies at colonies; Warzybok et al., 2018) and may be applied to develop spatially explicit layers of forage consumption through integrating bioenergetics, aggregation occurrence, and forage species availability (Vasbinder et al., 2023). Within the CCE, understanding the factors that drive predation of juvenile Chinook salmon (*Oncorhynchus tshawytscha*) during early ocean emigration benefited from examining the interannual aggregation intensity and diet of common murre (Wells et al., 2017). The daily predictions of common murre aggregation distribution developed in this study could be examined as a dynamical layer in mechanistic models of juvenile salmon predation (Vasbinder et al., 2023).

### Implications for ecosystem and fisheries monitoring

Although forage species were not explicitly considered as covariates in the species aggregation model, our observational record permits an examination of the forage species that likely support the modeled aggregation occurrence patterns. For example, common murre and western gull aggregations occur largely on the continental shelf and are associated with increased concentrations of euphausiids (*Euphausia pacifica*, *Thysannoessa spinifera*), northern anchovy (*Engraulis mordax*), juvenile rockfish (*Sebastes* spp.), Pacific sanddab (*Citharichthys sordidus*), juvenile salmon (*Onchorynchus* spp.), and market squid (*Doryteuthis opalescens*). Black‐footed albatross and pink‐footed shearwater aggregations occurred more frequently offshore in outer slope waters, while sooty shearwater aggregations overlapped with slope and deep canyon habitats; these regions have increased concentrations of euphausiids (*E. pacifica*), sergestids, mesopelagic fish and squid species, and Pacific hake (*Merluccius productus*) (Santora et al., 2012). Northern anchovy and juvenile rockfish, two important forage fish species to seabird consumption and reproduction regionally (Warzybok et al., 2018), have distinctive spatial distribution patterns (Santora et al., 2021). During a strong recruitment year, the abundance of total juvenile rockfish are spatially consistent throughout the study area, while when regional anchovy populations increase in size, they may expand further offshore (Santora et al., 2014) or become highly concentrated nearshore during lower population sizes (Santora et al., 2020; Wells et al., 2017). Within this study region, temporal patterns of northern anchovy and juvenile rockfish are typically out of phase (though moderate years of abundance occur), with increases in juvenile rockfish (and krill) during cooler and stronger upwelling years (Ralston et al., 2015; Santora et al., 2014). Models predicted that occurrence of common murre and sooty shearwater aggregations, the two most abundant seabird foragers on anchovy, also increased during warm years.

Simulations of krill aggregation formation and persistence suggest that the occurrence, size, and intensity of aggregations increase during spring when conditions are cooler (Fiechter et al., 2020; Messié et al., 2022; Santora, Dorman, & Sydeman, 2017). We found that the occurrence of Cassin's auklet aggregations increased during cooler years, presumably in response to krill availability (Ainley et al., 1996). Overall, however, albatrosses, gulls, and pink‐footed shearwater also were predicted with a higher probability of occurrence during cooler and likely better krill availability years (Santora et al., 2014). Therefore, changes in ocean temperature and forage species availability likely modulate the different occurrence patterns of seabird species aggregations, and when used concurrently, such data may provide a useful ecosystem monitoring indicator.

Are warm or cool seas associated with an increased chance of seabird aggregations? We found that ocean climate plays a significant role in the structuring and spatial organization of seabird aggregations. When pelagic species alter their aggregation intensity, including size and distribution, (i.e., aggregate closer to shore), there is often greater potential for interactions with coastally managed species and human activities (e.g., salmon predation, fisheries bycatch; Wells et al., 2017; Santora et al., 2020). Species aggregation occurrence in spring was significantly associated with lagged SST and SLA during winter though spring, indicating that relative species aggregation occurrence may be predictable as an “ecosystem state.” That is, during a warm winter, we may anticipate higher aggregation occurrence of murres and shearwaters during the following spring and potential associated ecosystem consequences (e.g., concentrations nearshore, predation of protected salmon species). Seasonal to annual forecasts of global and regional SST products may be applied to predict aggregation occurrence for mitigating adverse effects on protected species (Adams et al., 2012; Brodie et al., 2023).

### Implications for conservation and management

Although seabirds are global sentinels for ecosystem monitoring (Cairns, 1987), it is difficult to fully account for the conservation needs of the species examined in the present analysis. However, highlighting a few examples may shed light on the potential application of aggregation models to underscore the importance of essential foraging habitat. Two species are listed as “Near‐threatened” (Cassin's auklet and sooty shearwater) and two are “Vulnerable” (pink‐footed shearwater and black‐footed albatross)—therefore, knowledge of where, when, and how persistently these species aggregate throughout their life cycle is important for conservation planning.

#### Fishery bycatch

All seabird species have had negative interactions with fisheries in some form or another (i.e., bycatch, prey reductions). Regionally, we must acknowledge the high mortality that the common murre population experienced from gillnets and other sources of entanglement, and significant bycatch of albatrosses and shearwaters, both near their colonies and in the North Pacific (Ainley & Lewis, 1974; Arata et al., 2009; Carle et al., 2019; Forney et al., 2001; Fox et al., 2021; Gladics et al., 2017). Interactions between fisheries and seabirds occur at the scale of aggregations (Joo et al., 2014; Mangel, 1994). One of the most apparent seabird aggregation patterns at sea is certain species' response to the presence and activities of fishing vessels (e.g., drift gillnet, long‐lining, catching and processing vessels; Ainley et al., 1981; Gladics et al., 2017; Jannot et al., 2021; Melvin et al., 1999). Response to fishery discards, setting of long‐lining gear, and haul back events by purse‐seiners, if not properly conducted with technology and practices to reduce negative interactions, scores of seabirds may be taken as bycatch (Melvin et al., 1999; Gladics et al., 2017; Jannot et al., 2021). Our predictions of aggregation occurrence were entirely environmentally driven, so although they highlight important habitat for supporting aggregations, they did not include the behavioral dynamics that could help understand how they respond to the presence of interspecific species (e.g., whales) and human activities that influence aggregation formation. Our aggregation modeling approach did not include the presence or activity of fishing vessels, but it could be modified to include P/A and behavior of fishing vessels, perhaps through combining with individual‐based models, trained using vessel‐tracking databases (Joo et al., 2014). Regionally within the CCE, a combined aggregation occurrence and behavioral model could be implemented to explore how assessment of shifts in the intensity and activities of the market squid, Chinook salmon, and albacore tuna fisheries could improve estimates of resource clustering used by these fisheries and interactions with their predators.

#### Marine renewable energy

Utilization of seabird aggregation models may be useful for informing decisions to benefit conservation and biodiversity monitoring. Collectively, all available and robust SDM output developed from occurrence records derived from an ecosystem monitoring survey has potential to guide adaptive survey planning and support conservation priorities (i.e., identify critical location for organisms) (Sofaer et al., 2019). Regarding future adaptive survey monitoring of seabirds combined with future projections using climate models, we can assess how ocean habitat use supporting important seabird aggregation areas will change or potentially shift due to climate change and variability. More prescient, development of marine renewable energy is increasing, requiring assessments and applications of SDMs to evaluate potential conflicts with marine wildlife, especially protected species. Conservation conflicts and planning between offshore wind energy and seabirds are nothing new (Furness et al., 2016), and substantial research has addressed seabird vulnerability to wind farms under a variety of conditions, though not within eastern boundary currents (e.g., collisions, migration timing, reduction in foraging habitat; Garthe et al., 2023; Peschko et al., 2020). Regarding impacts of offshore windfarms to seabird collision risk (or avoidance), the aggregation model may allow us to examine or understand what environmental conditions would likely facilitate increased occurrence of aggregations within the vicinity of windfarm areas. Simply knowing that aggregations of particular species, especially those that may act as a catalyst for extracting other less abundant or rare species (e.g., shearwater and murre flocks), increases during warmer ocean conditions, should be relevant information for minimization of impacts from human disturbances. Given that seabird aggregation behavior is an important functional trait of seabird species, evaluating how their aggregations may change due to the location of windfarms should be a conservation concern.

#### Water quality and disease

A stimulus for this study was indeed the documentation of the strange shearwater fallout coinciding with the harmful algal bloom (HAB) event off central California in 1961 (Bargu et al., 2012). Seabirds are susceptible to toxic poisoning from HABs, through direct interaction with waters within blooms, but mostly through consumption of prey that have accumulated the toxin (e.g., anchovy, krill; Bargu et al., 2012; Bernstein et al., 2021; Ryan et al., 2017). The occurrence and persistence of HABs likely increase during ocean warming events (Ryan et al., 2017), especially during heatwaves, and have radiating effects on the marine food web and fisheries (Cavole et al., 2016). Therefore, an interaction between the patchiness and persistence of HABs and seabirds likely exists, and those species that increase their aggregation occurrence during warm years may be disproportionately affected compared to species more likely to aggregate during cool years. Furthermore, increased disease transmission may occur and impact seabird populations differently for those that form denser breeding colonies and aggregations at sea. For example, waterfowl aggregations have been linked to increased prevalence and susceptibility to disease due to higher transmission rates occurring with larger aggregations (Gaidet et al., 2012; Newman et al., 2007). Currently, the High Pathogenicity Avian Influenza virus is already impacting many seabird populations severely and could be further exacerbated for those species that routinely form dense aggregations on land and sea (Caliendo et al., 2022).

#### Oil spills

There is a long history of oil spills, big and small, having impacts on the marine ecosystem and seabirds within the central and southern California Current (Carter et al., 2003). Given their life on the surface of the ocean, seabirds are especially susceptible to oil spills, usually resulting in significant mortality events; if not quickly treated, oiled seabirds perish quickly. Ocean currents and conditions play a critical role in the dispersion and concentration of crude oil spills (De Dominicis et al., 2016), which in a way may potentially mirror the environmental drivers of seabird aggregations developed in this study (Fraser et al., 2022). An obvious application of the seabird aggregation model may be made through utilization of scenarios based on historical oil spills to prepare for contemporary and future spills. For example, if an oil spill occurred during a warm (or cool) ocean period, then we might anticipate that some of the species we investigated could be more at risk of significant oiling and ultimately death. Knowing that murres and shearwaters are more likely to occur in aggregations during a warm period that overlaps with an oil spill event could aid response teams aiming to collect and clean oiled seabirds. Further, species that aggregate near shore or within shipping lanes may be at greater risk of oiling (e.g., murres, gulls; Cimino et al., 2022). Our aggregation model is set up to run daily predictions from April–June, and future effort could operationalize the model as a research and conservation tool to aid in seabird recovery during. This could be examined through reanalysis of daily aggregation patterns and to identify the location of persistent areas of aggregation, knowledge of which could prepare recovery effort during responses to oil events and assess mortalities involving estimates of aggregation detection probability (Fraser et al., 2022). Lastly, given the significant lagged correlations between spring species aggregation occurrence and either SST and SLA back to winter, the model may be useful for tracking the potential impact of an oil spill event that occurred in winter or spring, potentially serving as an early warning system.

## CONFLICT OF INTEREST STATEMENT

The authors declare no conflicts of interest.

## Supporting information


Appendix S1.

## Data Availability

Data and code (Suca, 2025) are available on Zenodo at https://doi.org/10.5281/zenodo.15459936. Sea level anomaly satellite data (Copernicus Climate Change Service [C3S] Climate Data Store, 2018) are available at https://doi.org/10.24381/cds.4c328c78. National Oceanographic and Atmospheric Administration (NOAA) optimum interpolation sea surface temperature satellite data (Huang et al., 2020) are available from NOAA National Centers for Environmental Information at https://doi.org/10.25921/RE9P-PT57.
