# Supplementary material for: Species aggregation models resolve essential foraging habitat: Implications for conservation and management
Source: Ecol Appl. 2025 Jul 3;35(5):e70068. doi: 10.1002/eap.70068 (PMC12223939; doi:10.1002/eap.70068)
Supplement: Supplementary file 1 — Appendix S1. [file EAP-35-e70068-s001.pdf]

Appendix S1

Journal: Ecological Applications

Title: Species aggregation models resolve essential foraging habitat: Implications for conservation and management

Authors: Jarrod A. Santora, Justin J. Suca, Megan Cimino, Elliott L. Hazen, John C. Field, Steven J. Bograd, Brian K. Wells, Isaac D. Schroeder

Appendix S1: Table S1 (a-c)

(a) Cassin's Auklet

Response	CV Method	Mean AUC	Mean TSS
P/A	75/25	0.855 (0.813-0.889)	0.569(0.489-0.658)
Aggregation	75/25	0.849(0.742-0.956)	0.627(0.454-0.818)
P/A	LOYO	0.673(0.518-0.807)	0.320 (0.151-0.524)
Aggregation	LOYO	0.478(0.051-1.0)	0.374(0.034-1)
Aggregation (Hurdle)	All Data	0.917 (0.873-0.947)	0.697 (0.605-0.812)

(b) Common Murre

Response	CV Method	Mean AUC	Mean TSS
P/A	75/25	0.883(0.855-0.903)	0.630(0.562-0.679)
Aggregation	75/25	0.823(0.728-0.885)	0.536(0.365-0.659)
P/A	LOYO	0.781(0.649-0.890)	0.487(0.258-0.709)
Aggregation	LOYO	0.620(0.321-0.903)	0.480(0.103-0.840)
Aggregation (Hurdle)	All Data	0.903 (0.885-0.919)	0.657 (0.594-0.701)

(c) Western Gull

Response	CV Method	Mean AUC	Mean TSS
P/A	75/25	0.777 (0.717-0.834)	0.425(0.333-0.507)
Aggregation	75/25	0.850(0.739-0.965)	0.610(0.481-0.876)
P/A	LOYO	0.649(0.521-0.755)	0.295(0.116-0.467)
Aggregation	LOYO	0.623(0.167-1.0)	0.449(0.017-1.00)
Aggregation (Hurdle)	All Data	0.881 (0.839-0.904)	0.628 (0.543-0.695)

Appendix S1: Table S1: Summary statistics for assessment of model performance: (a) Cassin's auklet, (b) Common Murre, and (c) Western Gull. Response is model type, P/A is presence/absence, CV is cross-validation method, AUC is area under curve (threshold independent measure of accuracy) and TSS is true skill statistic (threshold dependent measure of accuracy) and LOYO is leave one year out. See Methods for description of model performance assessment.

Appendix S1: Table S1 (d-f)

(d) Sooty Shearwater

Response	CV Method	Mean AUC	Mean TSS
P/A	75/25	0.826 (0.783-0.864)	0.515(0.440-0.589)
Aggregation	75/25	0.850 (0.766-0.940)	0.603 (0.459-0.782)
P/A	LOYO	0.603(0.503-0.805)	0.249 (0.087-0.503)
Aggregation	LOYO	0.627(0.195-0.99)	0.434(0.142-0.923)
Aggregation (Hurdle)	All Data	0.892(0.860-0.916)	0.629(0.575-0.685)

(e) Pink-footed Shearwater

Response	CV Method	Mean AUC	Mean TSS
P/A	75/25	0.790 (0.711-0.873)	0.452(0.299-0.589)
Aggregation	75/25	0.893(0.738-0.985)	0.714(0.412-0.957)
P/A	LOYO	0.609(0.480-0.809)	0.282 (0.104-0.603)
Aggregation	LOYO	0.506(0.25-1.00)	0.412(0.00-1.00)
Aggregation (Hurdle)	All Data	0.928 (0.881-0.958)	0.745 (0.645-0.823)

(f) Black-footed Albatross

Response	CV Method	Mean AUC	Mean TSS
P/A	75/25	0.830 (0.769-0.883)	0.518(0.411-0.654)
Aggregation	75/25	0.800(0.667-0.909)	0.525(0.346-0.719)
P/A	LOYO	0.719(0.613-0.917)	0.389(0.225-0.760)
Aggregation	LOYO	0.517(0.235-0.936)	0.339(0.00-0.936)
Aggregation (Hurdle)	All Data	0.842 (0.820-0.865)	0.554 (0.483-0.618)

Appendix S1: Table S1 (d-f): Summary statistics for assessment of model performance: (d) Sooty Shearwater, (b) Pink-footed shearwater, and (c) Black-footed albatross. Response is model type, P/A is presence/absence, CV is cross-validation method, AUC is area under curve (threshold independent measure of accuracy) and TSS is true skill statistic (threshold dependent measure of accuracy) and LOYO is leave one year out. See Methods for description of model performance assessment.

Appendix S1: Figure S1 (a)

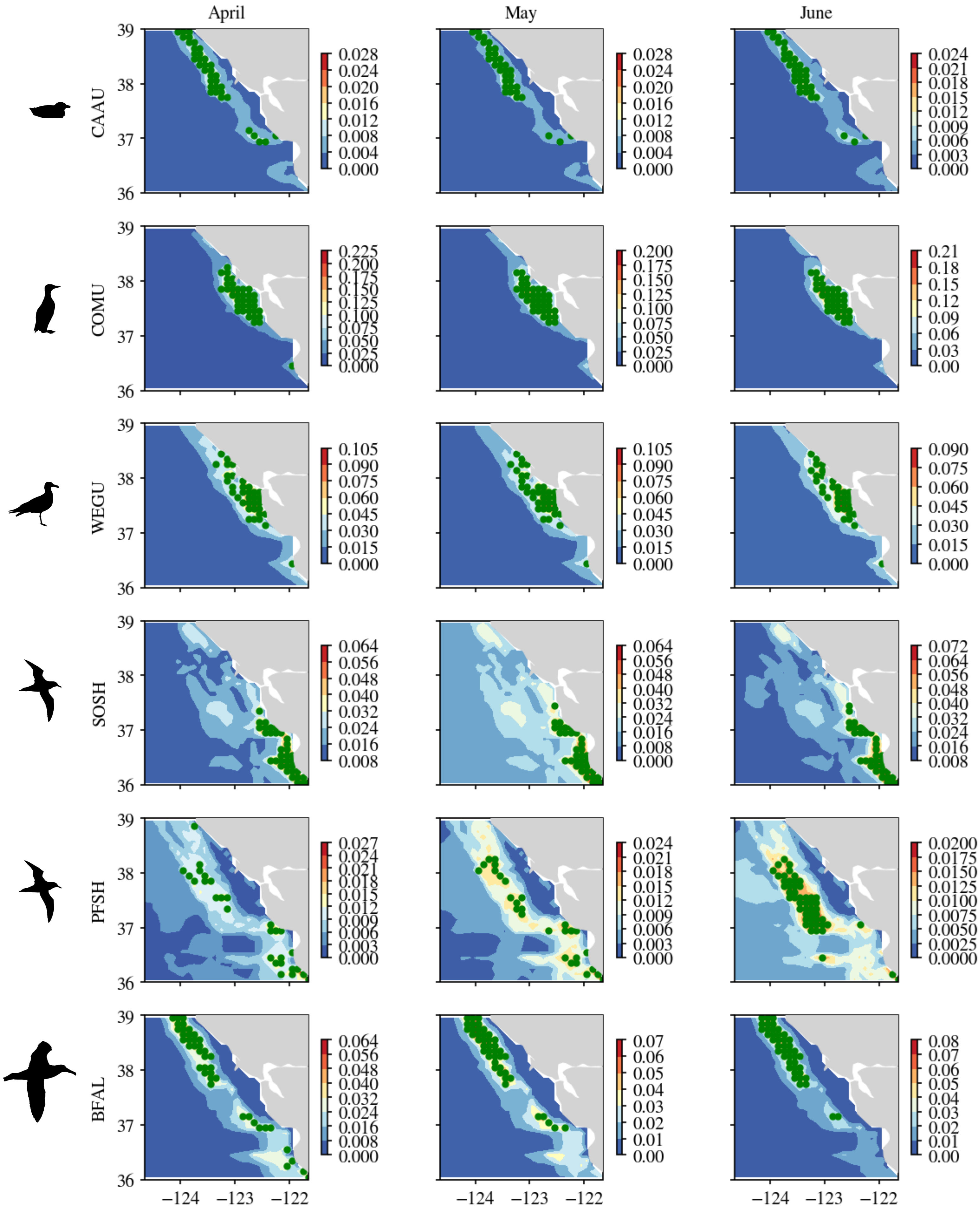

Appendix S1: Figure S1 (a): Threshold analysis of aggregation probability based on predicted spatial mean patterns for April, May and June. Green dots indicate grid cells where the threshold (greater than 2 standard deviations) occurred, indicating where aggregations were most likely to occur. CAAU Cassin's Auklet, COMU Common murre, WEGU Western Gull, SOSH, Sooty Shearwater, PFSH Pink-footed shearwater, BFAL Black-footed Albatross. Taxa silhouettes for common murre, Cassin's auklet were drawn by the author (Jarrod Santora). Silhouettes for the albatross (Alexandre Vong) and the gull and shearwaters (Juan Carlos Jerí) are derived from www.phylopic.org under a CC0 1.0 Universal Public Domain Dedication license (https://creativecommons.org/publicdomain/zero/1.0/).

Appendix S1: Figure S1 (b)

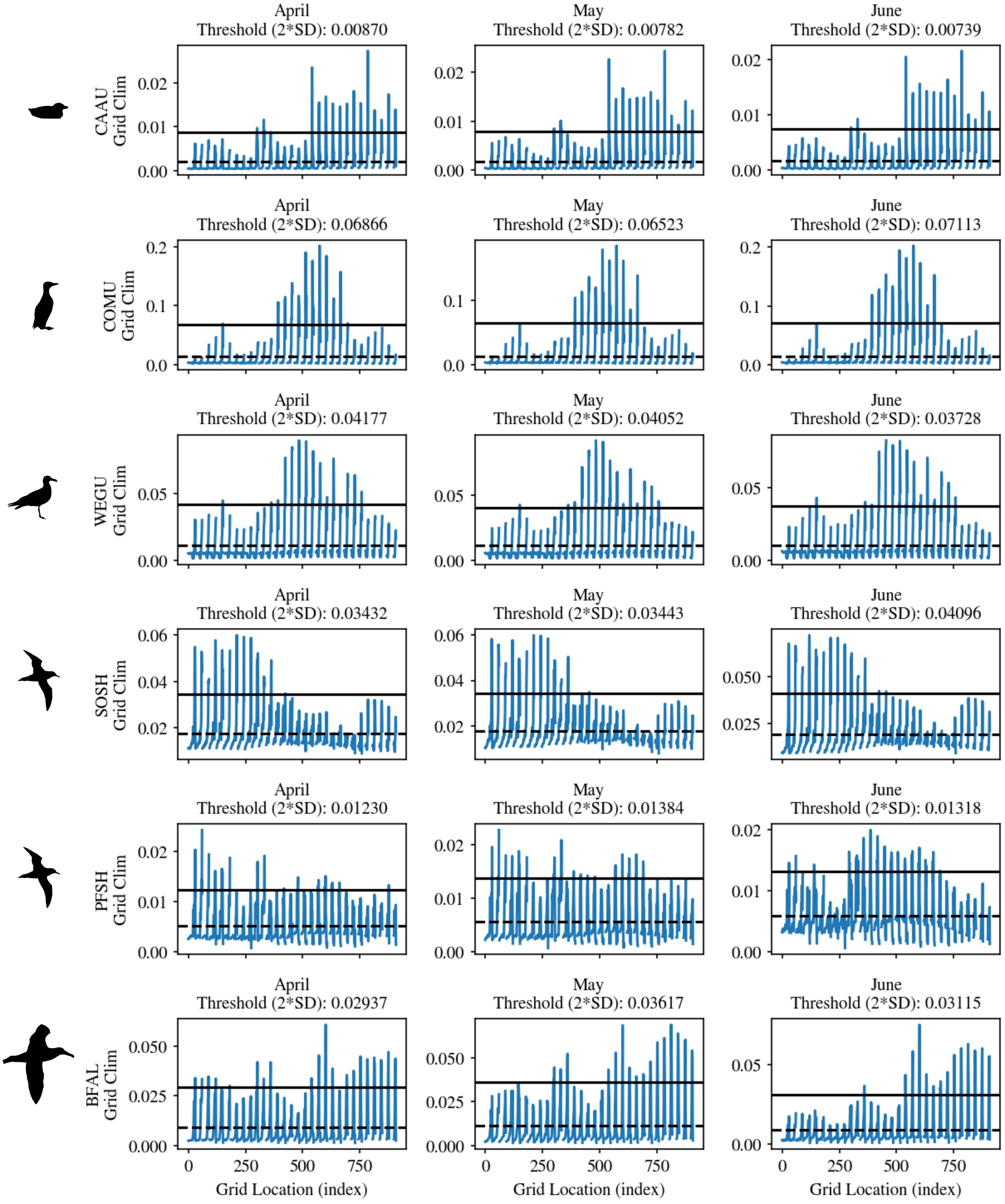

Appendix S1: Figure S1 (b): Threshold analysis of aggregation probability based on predicted spatial mean patterns for April, May and June. X-axis is an index of grid location and y-axis is the probability per grid cell; dashed line is the mean and the dark line is the threshold of 2 standard deviations. CAAU Cassin's Auklet, COMU Common murre, WEGU Western Gull, SOSH, Sooty Shearwater, PFSH Pink-footed shearwater, BFAL Black-footed Albatross. Taxa silhouettes for common murre, Cassin's auklet were drawn by the author (Jarrod Santora). Silhouettes for the albatross (Alexandre Vong) and the gull and shearwaters (Juan Carlos Jerí) are derived from www.phylopic.org under a CC0 1.0 Universal Public Domain Dedication license (https://creativecommons.org/publicdomain/zero/1.0/).

Appendix S1: Figure S2

Cassin’s auklet

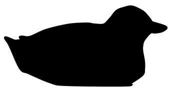

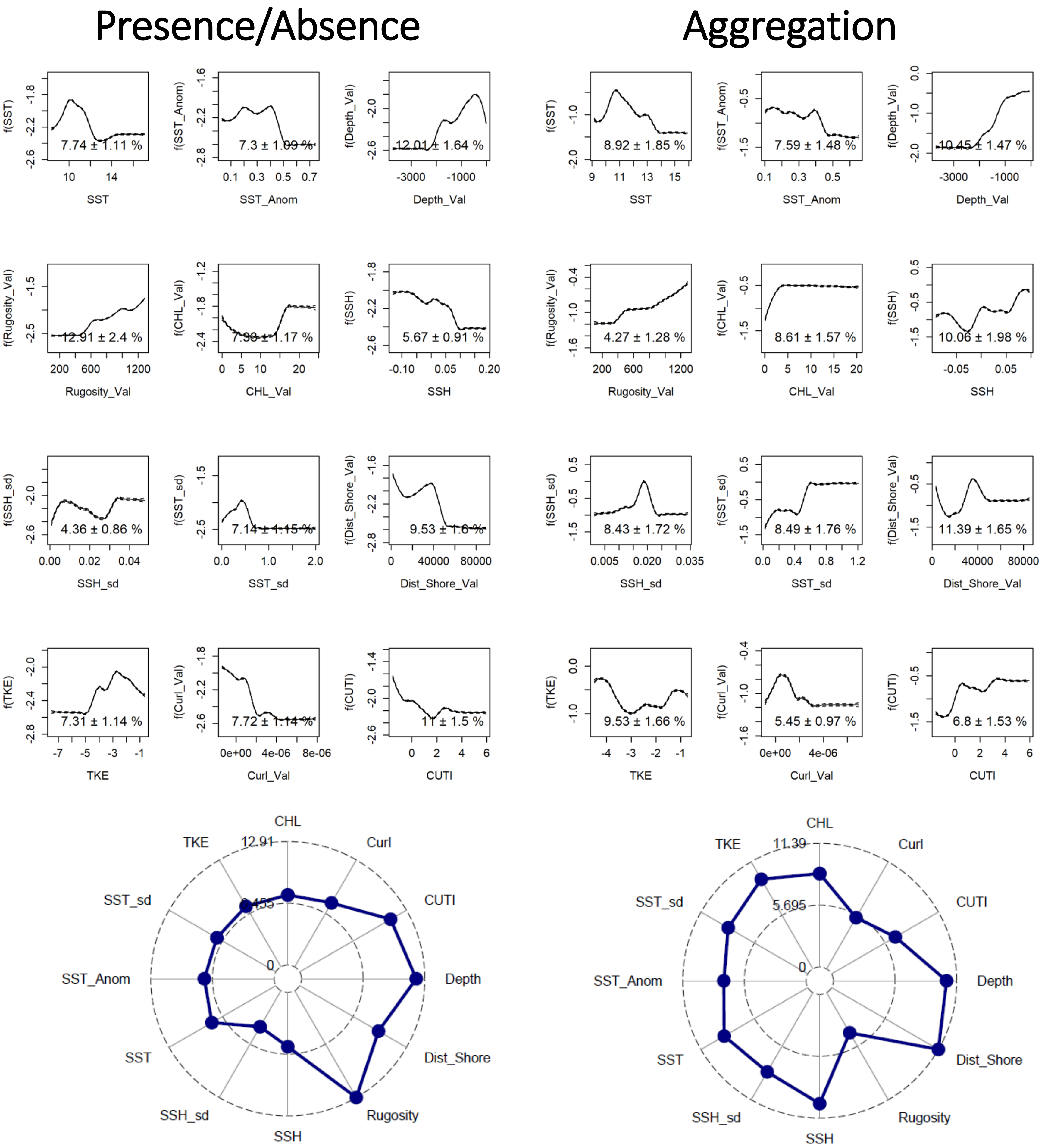

Appendix S1: Figure S2: (top) Partial dependence plots for covariates included in the Species Distribution Aggregation Model (SADM) for Cassin’s Auklet. (bottom) corresponding radar plots for relative importance of model covariates. See Figure 1 for covariate descriptions. Values on partial dependence plots indicate relative importance of the covariate and is summarized on the radar plots. Cassin’s auklet silhouette was drawn by the author (Jarrod Santora).

Appendix S1: Figure S3

Common murre

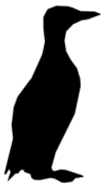

Presence/Absence

Aggregation

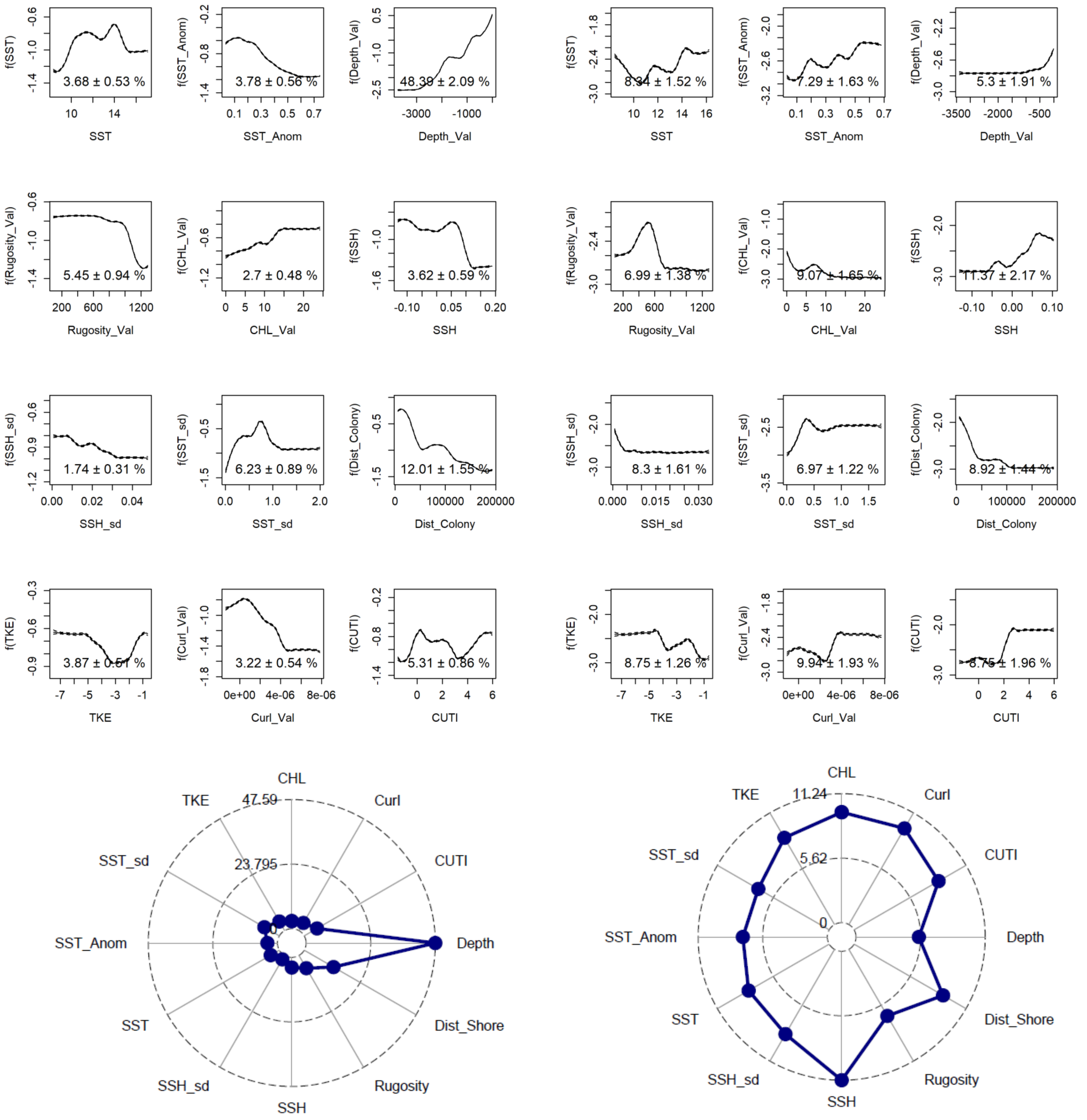

Appendix S1: Figure S3: (top) Partial dependence plots for covariates included in the Species Distribution Aggregation Model (SADM) for Common Murre. (bottom) corresponding radar plots for relative importance of model covariates. See Figure 1 for covariate descriptions. Values on partial dependence plots indicate relative importance of the covariate and is summarized on the radar plots. Taxa silhouette for the common murre was drawn by the author (Jarrod Santora).

Appendix S1: Figure S4

Western gull

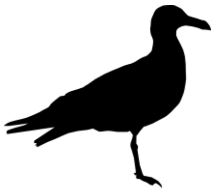

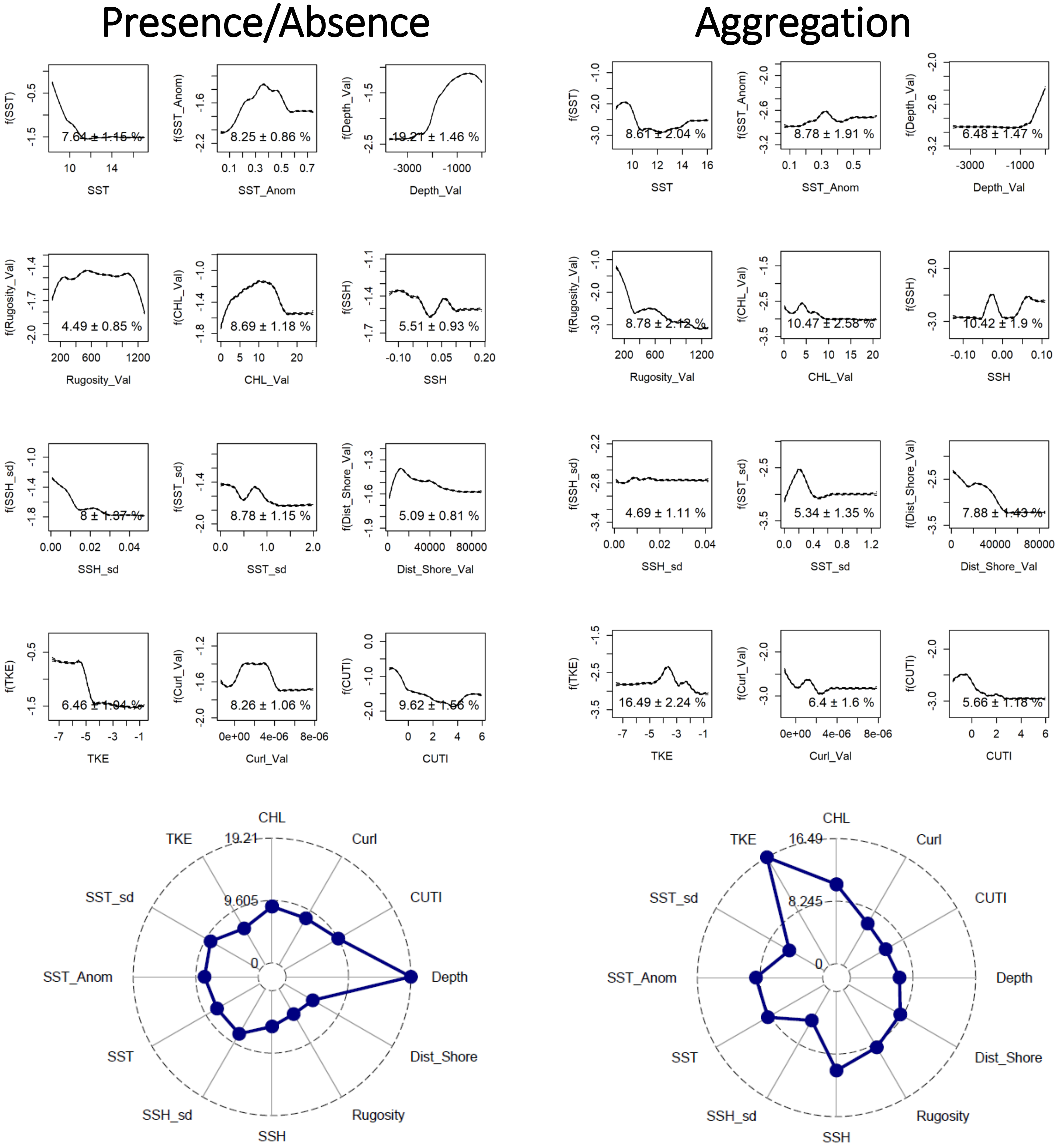

Appendix S1: Figure S4: (top) Partial dependence plots for covariates included in the Species Distribution Aggregation Model (SADM) for Western Gull. (bottom) corresponding radar plots for relative importance of model covariates. See Figure 1 for covariate descriptions. Values on partial dependence plots indicate relative importance of the covariate and is summarized on the radar plots. The silhouette for the gull is derived from www.phylopic.org (Juan Carlos Jerí) under a CC0 1.0 Universal Public Domain Dedication license (https://creativecommons.org/publicdomain/zero/1.0/).

Appendix S1: Figure S5

Sooty shearwater

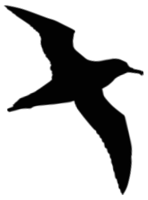

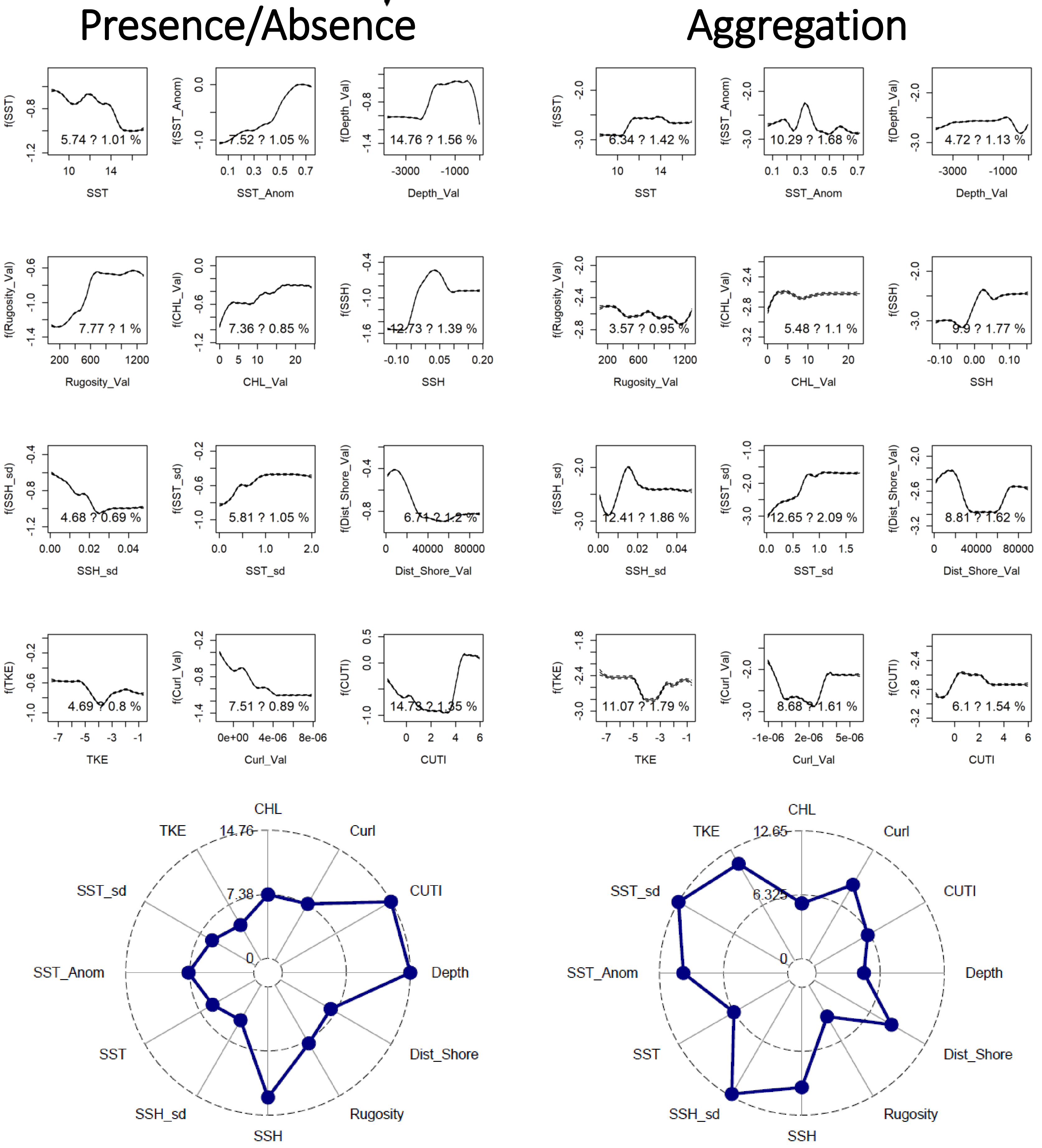

Appendix S1: Figure S5: (top) Partial dependence plots for covariates included in the Species Distribution Aggregation Model (SADM) for Sooty shearwater. (bottom) corresponding radar plots for relative importance of model covariates. See Figure 1 for covariate descriptions. Values on partial dependence plots indicate relative importance of the covariate and is summarized on the radar plots. The silhouette for the shearwater is derived from www.phylopic.org (Juan Carlos Jerí) under a CC0 1.0 Universal Public Domain Dedication license (https://creativecommons.org/publicdomain/zero/1.0/).

Appendix S1: Figure S6

Pink-footed shearwater

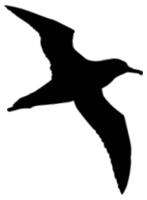

Presence/Absence

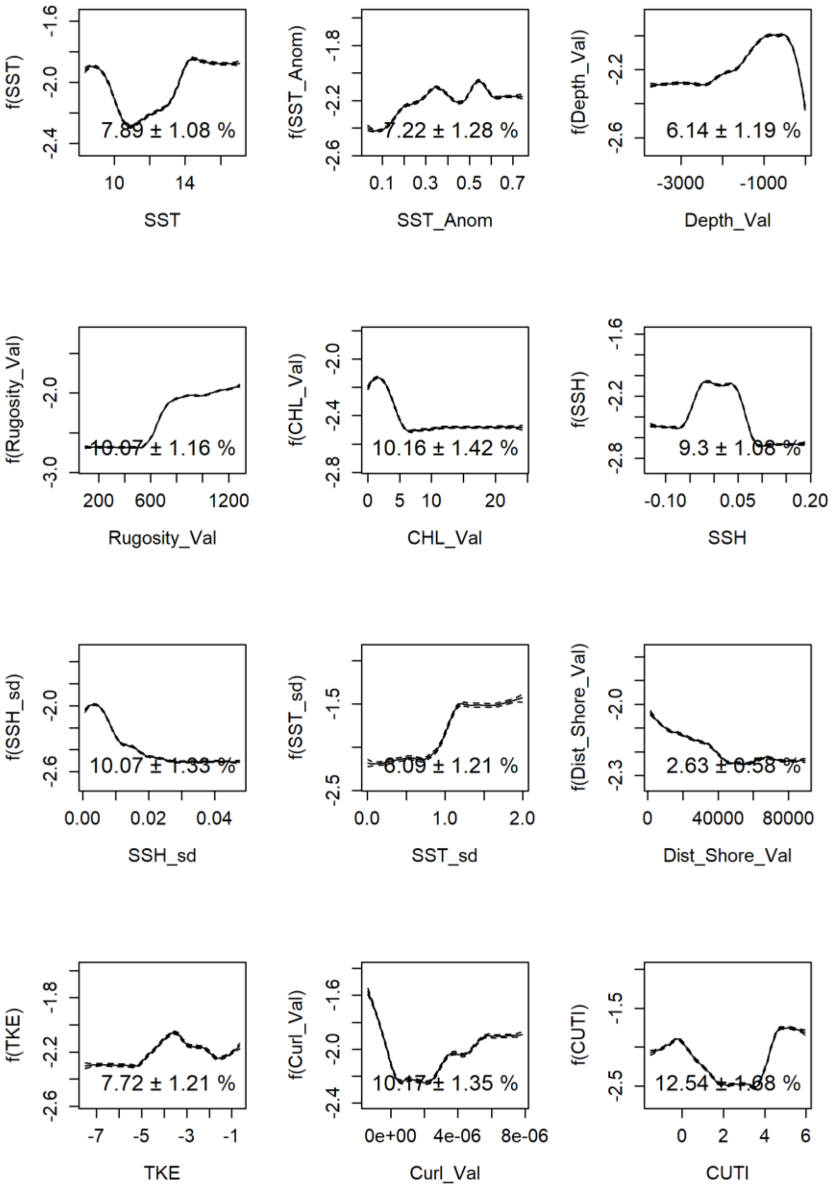

Aggregation

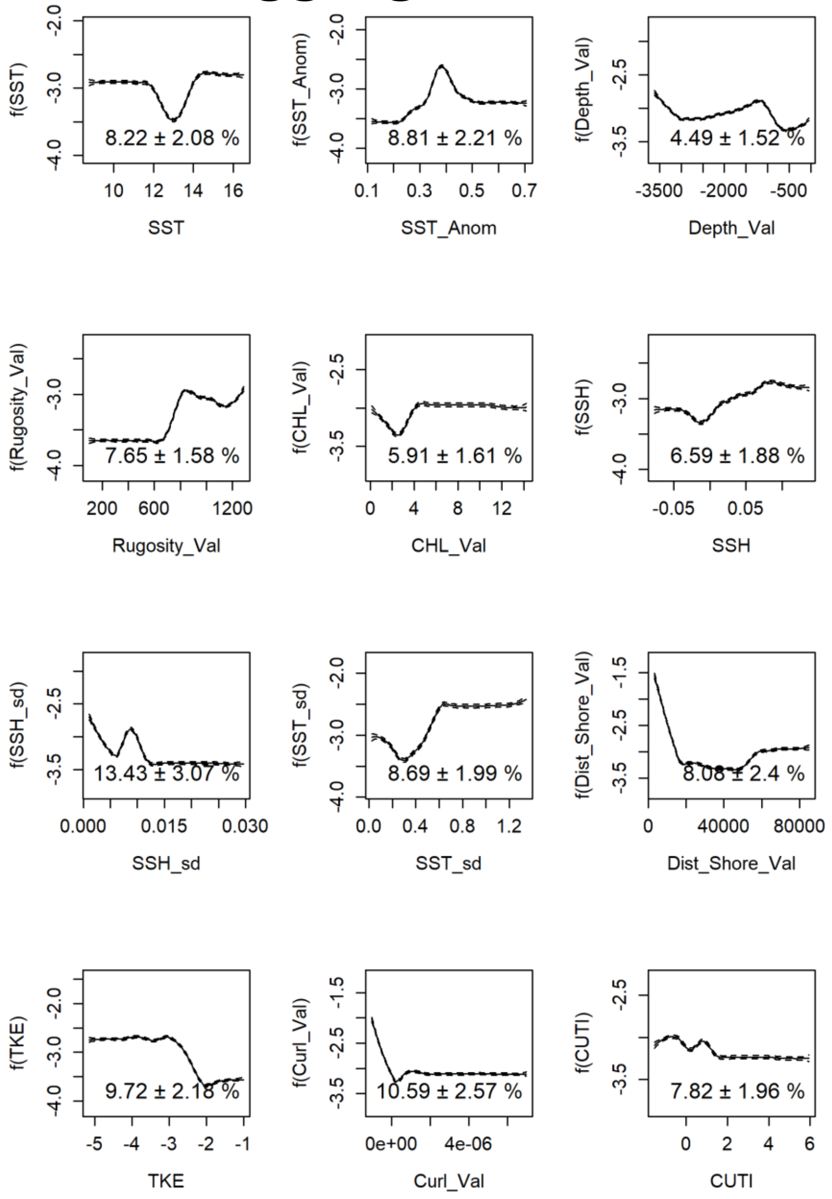

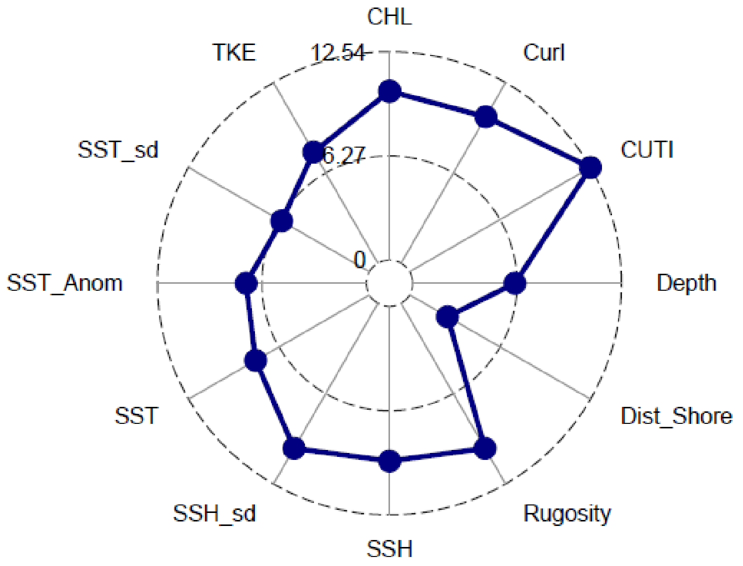

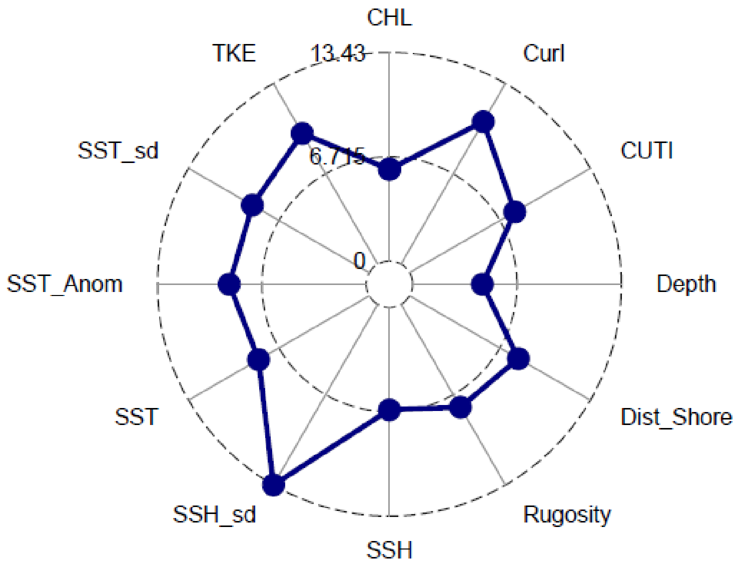

Appendix S1: Figure S6: (top) Partial dependence plots for covariates included in the Species Distribution Aggregation Model (SADM) for Pink-footed shearwater. (bottom) corresponding radar plots for relative importance of model covariates. See Figure 1 for covariate descriptions. Values on partial dependence plots indicate relative importance of the covariate and is summarized on the radar plots. The silhouette for the shearwater is derived from www.phylopic.org (Juan Carlos Jerí) under a CC0 1.0 Universal Public Domain Dedication license (https://creativecommons.org/publicdomain/zero/1.0/).

Appendix S1: Figure S7

Black-footed albatross

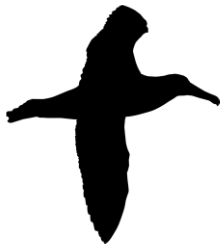

Presence/Absence

Aggregation

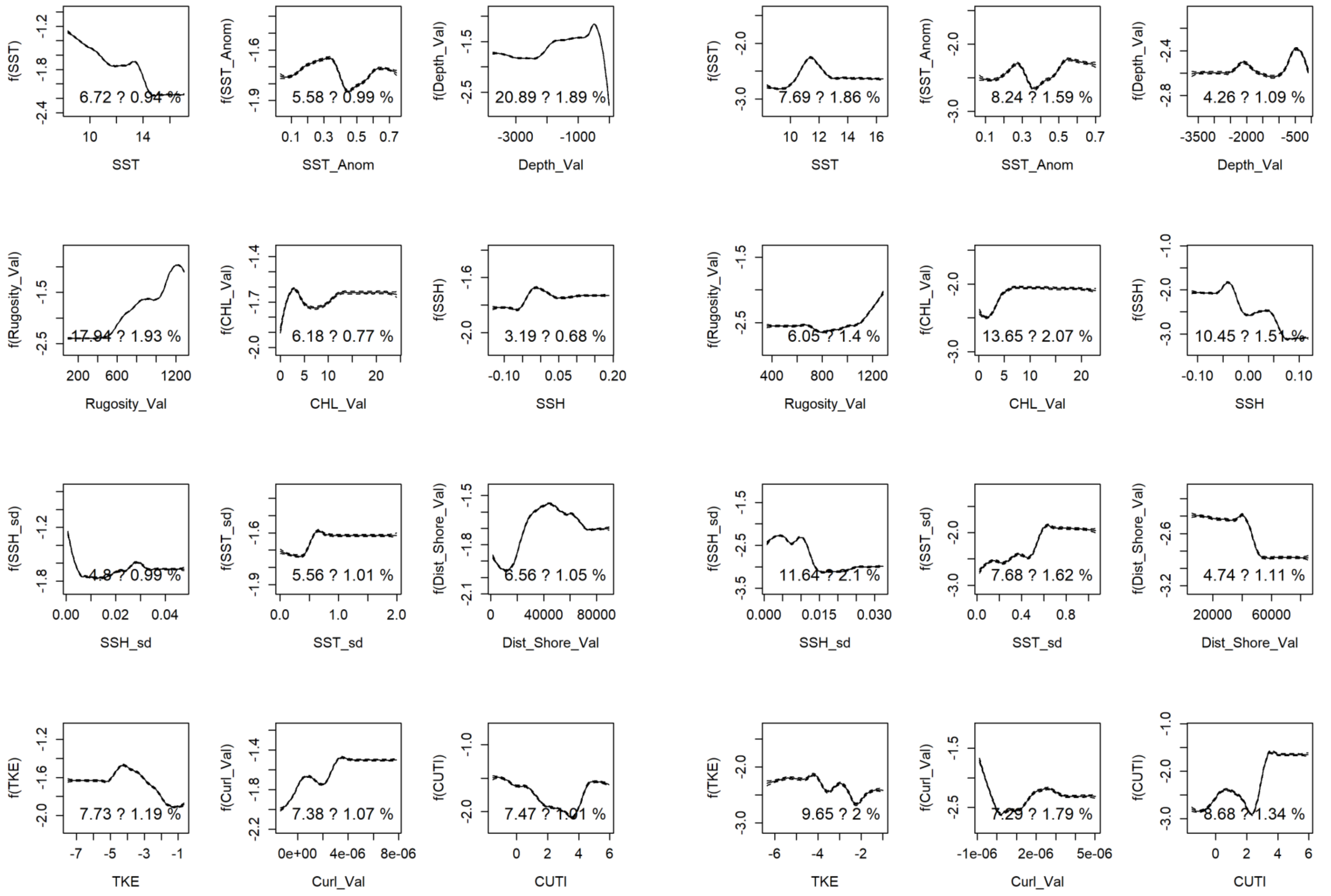

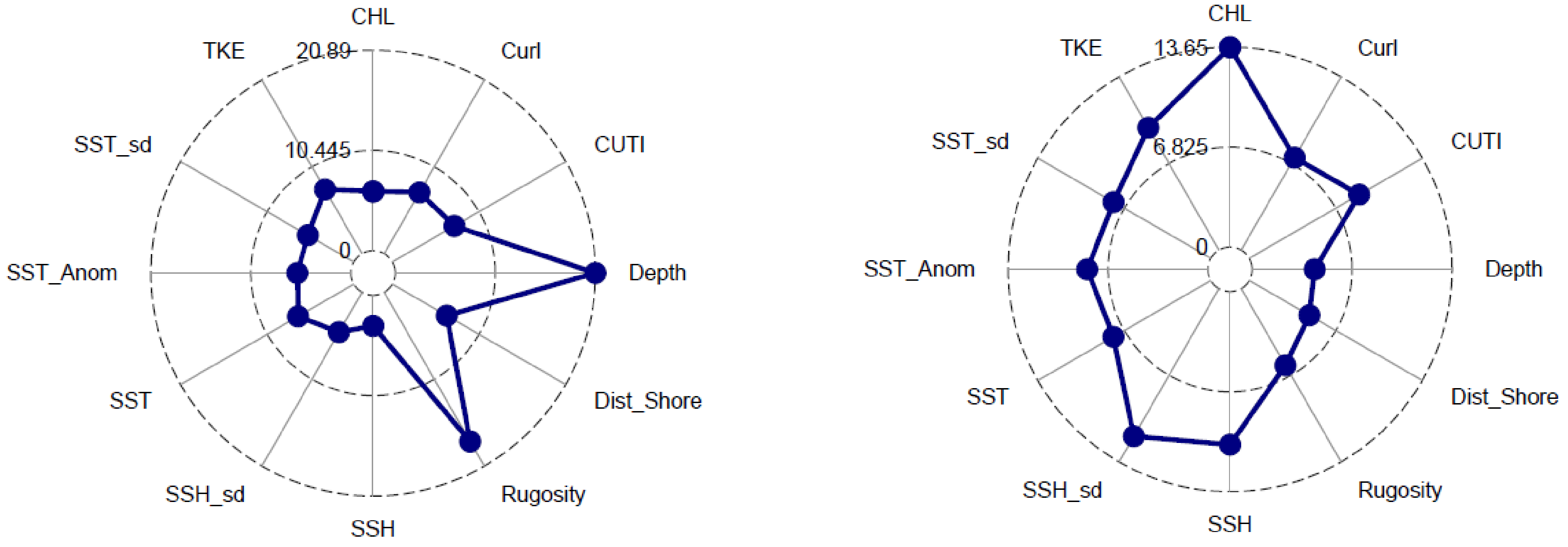

Appendix S1: Figure S7: (top) Partial dependence plots for covariates included in the Species Distribution Aggregation Model (SADM) for Black-footed Albatross. (bottom) corresponding radar plots for relative importance of model covariates. See Figure 1 for covariate descriptions. Values on partial dependence plots indicate relative importance of the covariate and is summarized on the radar plots. The silhouette for the albatross is derived from www.phylopic.org (Alexandre Vong) under a CC0 1.0 Universal Public Domain Dedication license (https://creativecommons.org/publicdomain/zero/1.0/).

Appendix S1: Figure S8

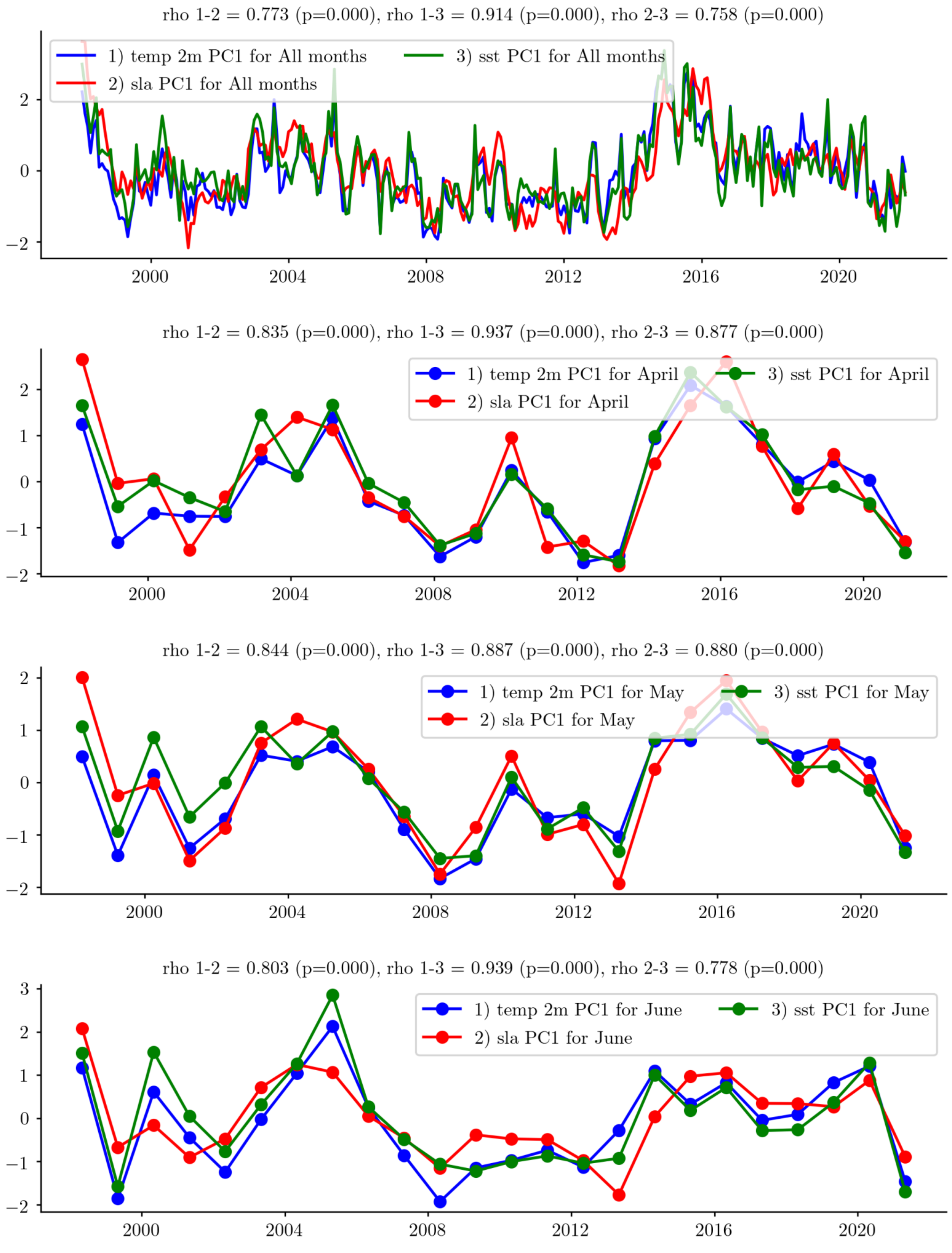

Appendix S1: Figure S8: Correlation between model covariates derived from ROMS and independent satellite data for sea surface temperature (SST; temp 2m) and sea level anomaly (SLA). Time series are from normalized EOFs; first principal components (PC1). Spearman rank correlations (rho) are shown for correlations between each (1-3; see inset legend) and are all significant at $p<0.001$.
